# How Universal Is the Relationship between Remotely Sensed Vegetation Indices and Crop Leaf Area Index? A Global Assessment

**DOI:** 10.3390/rs8070597

**Published:** 2016-07-15

**Authors:** Yanghui Kang, Mutlu Özdoğan, Samuel C. Zipper, Miguel O. Román, Jeff Walker, Suk Young Hong, Michael Marshall, Vincenzo Magliulo, José Moreno, Luis Alonso, Akira Miyata, Bruce Kimball, Steven P. Loheide

**Affiliations:** 1 Nelson Institute Center for Sustainability and the Global Environment; University of Wisconsin-Madison, 1710 University Avenue, Madison, WI 53726, USA; 2 Department of Geography, University of Wisconsin-Madison, Madison, WI post code, USA; 3 Department of Civil and Environmental Engineering, University of Wisconsin-Madison, Madison, WI post code, USA; 4 Terrestrial Information Systems Laboratory, NASA Goddard Space Flight Center, Code 619 Bld-32 S-036F, Greenbelt, MD post code, USA; 5 Department of Civil Engineering, Monash University, Clayton, Victoria post code, Australia; 6 Department of Agricultural Environment, National Academy of Agricultural Science (NAAS), RDA, Suwon post code, Korea; 7 Climate Change, Agriculture and Food Security Program, World Agroforestry Center, United Nations Ave, Nairobi post code, Kenya; 8 CNR –ISAFOM, Institute for Mediterranean Agricultural and Forest Systems, National Research Council, via Patacca 85, 80040 Ercolano (Napoli), Italy; 9 Institute of Biometeorology of the National Research Council (IBIMET-CNR), Firenze post code, Italy; 10 Laboratory for Earth Observation, Department of Earth Physics and Thermodynamics, University of Valencia, Burjassot, Valencia post code, Spain; 11 National Institute for Agro-Environmental Sciences, Tsukuba 305-0864, Japan; 12 US Arid-Land Agricultural Research Center, USDA, Agricultural Research Service, Maricopa, AZ post code, USA

**Keywords:** LAI, Vegetation Index, agriculture, landsat, agroecosystem modeling

## Abstract

Leaf Area Index (LAI) is a key variable that bridges remote sensing observations to the quantification of agroecosystem processes. In this study, we assessed the universality of the relationships between crop LAI and remotely sensed Vegetation Indices (VIs). We first compiled a global dataset of 1459 in situ quality-controlled crop LAI measurements and collected Landsat satellite images to derive five different VIs including Simple Ratio (SR), Normalized Difference Vegetation Index (NDVI), two versions of the Enhanced Vegetation Index (EVI and EVI2), and Green Chlorophyll Index (CI_Green_). Based on this dataset, we developed global LAI-VI relationships for each crop type and VI using symbolic regression and Theil-Sen (TS) robust estimator. Results suggest that the global LAI-VI relationships are statistically significant, crop-specific, and mostly non-linear. These relationships explain more than half of the total variance in ground LAI observations (*R*^2^ >0.5), and provide LAI estimates with RMSE below 1.2 m^2^/m^2^. Among the five VIs, EVI/EVI2 are the most effective, and the crop-specific LAI-EVI and LAI-EVI2 relationships constructed by TS, are robust when tested by three independent validation datasets of varied spatial scales. While the heterogeneity of agricultural landscapes leads to a diverse set of local LAI-VI relationships, the relationships provided here represent global universality on an average basis, allowing the generation of large-scale spatial-explicit LAI maps. This study contributes to the operationalization of large-area crop modeling and, by extension, has relevance to both fundamental and applied agroecosystem research.

## 1. Introduction

Leaf Area Index (LAI), defined as one half the total green leaf area (double-sided) per unit horizontal ground surface area of vegetation canopy [1,2], is an essential biophysical variable used extensively in soil-vegetation-atmosphere modeling [3–5]. In agroecosystems, the total leaf area of the crop canopy, as quantified through LAI, is one of the key constraints on carbon assimilation and transpiration rates, which together drive the accumulation of crop primary productivity [6,7]. Therefore, LAI is commonly required to estimate photosynthesis, evapotranspiration, crop yield, and many other physiological processes in agroecosystem studies [8–11].

LAI has historically been measured for crop canopies using in situ (destructive or optical) approaches or remote sensing techniques [12–16]. Although the in situ approaches are accurate and easy to implement, they are also labor and time intensive, and the sample-based measurements are spatially discontinuous [17,18]. In contrast, remote sensors onboard satellite or aircraft are capable of making spatially complete measurements of surface reflectance, which are related to the greenness of canopy. Therefore, there has been continuous interest in estimating LAI using images acquired from airborne/space-borne sensors [19–26].

To estimate LAI using remotely sensed data, two types of methodologies have been adopted: the process-based approach and the empirical approach based on Vegetation Index (VI), also called the VI approach [24,27]. Process-based approaches obtain LAI estimates by inverting a radiative transfer model forced with canopy reflectance data retrieved remotely [28–33]. Radiative transfer models simulate the (bidirectional) reflectance of the land surface through a series of physical or mathematical description of the physical and radiometric properties of background (i.e., soil or snow surface), the object (i.e., canopy or other surfaces), atmosphere, as well as solar and sensor geometries [34–39]. However, unknown model variables usually outnumber reflectance observations, leaving model inversion unsolved or with multiple solutions—an issue referred to as the “ill-posed problem” [40]. Therefore, although the process-based approach benefits from detailed physical descriptions of the atmosphere-canopy-soil system, its robustness largely depends on the accuracy of model parameters, which has limited its applicability in large scale [41].

Unlike the process-based approach, the VI approach provides a simple yet effective alternative by establishing a statistical relationship between remotely sensed VIs and observed LAI values, hereafter referred as an LAI-VI relationship [19,42,43]. VIs are constructed from reflectance of two or more spectral bands, and can be used to estimate biophysical/biochemical characteristics of vegetation, such as LAI, biomass, and canopy chlorophyll content [44–47]. A number of VIs has been show to correlate well with LAI. The earliest attempts used VIs such as the Simple Ratio (SR) [48] and Normalized Difference Vegetation Index (NDVI) [49], which were designed to accentuate the difference between red and near-infrared (NIR) reflectance. More optimized VIs were later proposed with increased sensitivity to vegetation characteristics (e.g., LAI) and minimized effect from confounding factors (e.g., canopy geometry, soil, and atmosphere). These include Soil Adjusted Vegetation Index (SAVI) [50], Atmospherically Resistant Vegetation Index (ARVI) [51], Enhanced Vegetation Index (EVI) [52–54], Modified Triangular Vegetation Index (MTVI2) [55], Wide Dynamic Range Vegetation Index (WDRVI) [56], and EVI2 [57]. Besides different types of VI used, the LAI-VI relationships also take various mathematical forms or equations, such as linear, exponential, logarithm, or polynomial [58–60].

Numerous studies, mostly at local scales, have tested the VI approach and demonstrated its effectiveness at various locations around the world, using either field-measured or remotely sensed reflectance data [61–64]. Nevertheless, the LAI-VI relationship is not unique, particularly in agricultural settings, but rather represented by a family of equations as a function of the specific geographical, biological, and environmental setting of a study. As a result, the VI approach requires a new set of LAI measurements to be made at each new location, each time, and for each crop, rendering its application impractical over large areas or multiple time periods [65–67].

This “one place, one time, one equation” issue has been identified as a major limitation of the VI approach to map LAI with remotely sensed observations [27,36,59,68,69]. While our knowledge of the dependence of the LAI-VI relationship on plant/crop types or canopy geometry continues to advance [43,58–60,63,70,71], a number of questions remain. For example, to what extent can we spatially and temporally generalize the LAI-VI relationships? Can the variation caused by a number of environmental factors be controlled within an acceptable range? Is there a “one-size-fits-all” relationship that suits a global sample across different crop types and time periods? To answer these questions, we (1) synthesized a global dataset of in situ crop LAI measurements and remotely sensed VIs derived from Landsat Thematic Mapper (TM) and Enhanced Thematic Mapper Plus (ETM+) images; (2) established LAI-VI relationships for different crop types and VIs; and (3) evaluated the universality and diversity of these LAI-VI relationships. For consistency with the general LAI research community, we have followed the Committee on Earth Observation Satellites Land Product Validation (CEOS LPV) LAI protocol throughout the data collection, analysis, and evaluation stages [2].

## 2. Data and Methodology

### 2.1. LAI data Collection and Quality Control

We assembled a global dataset of in situ crop LAI measurements from a number of sources, including regional flux networks, research campaigns, peer-reviewed journals, sample data in crop models, and investigators who collected the LAI data. To be included in our dataset, a set of LAI measurements had to have: (1) accurate geographical location; (2) information on date of measurement, crop type, and method of measurement; (3) cloud-free Landsat images available at the measurement location, within 15 days of the measurement time; (4) ancillary information about the experimental design.

We then conducted a thorough data quality control, using four rules to identify and eliminate data with potential quality issues:

Rule 1: LAI values less than 0.1 m^2^/m^2^ or greater than 6 m^2^/m^2^ are beyond the prediction power of VIs and were thus eliminated (details see Section 3.2.4) [58–60].
Rule 2: At each site from which in situ data were obtained, we examined the local LAI-VI relationships and used auxiliary data to identify potentially low quality data with respect to remote sensing applications. Our study was based on the widely supported assumption that a statistically significant LAI-VI relationship will exist at a given site; the lack of a significant relationship, therefore, indicates potential in situ or satellite data quality issues. In addition, since our LAI definition is only restricted to green leaves, we were careful in checking the phenological stage when the LAI was measured in each site, and removed LAI measured in senescence stage when leaves were not green. When there is no specific information on the phenological stage, we checked the time series of both LAI and VIs to make sure that all observations stopped at or shortly after peak growth period.
Rule 3: Any crop type with a sample size less than 1% of the overall dataset was eliminated.
Rule 4: After Rules 1–3 were checked, we applied a binned interquartile range (IQR) approach to eliminate additional outliers. First, we grouped LAI into 0.5 m^2^/m^2^ bins. Within each bin, the LAI data were ranked according to corresponding NDVI values from the lowest to the highest, and the median, 25% quartile (Q1), and 75% quartile (Q3) were computed. Then outliers were defined as values below Q1 – 1.5IQR or above Q3 + 1.5IQR.

Since LAI beyond 6 m^2^/m^2^ is not uncommon for crops like maize, wheat, and rice [36,65,125,135], we produced another version of dataset with slightly different quality control measures, where rule 1 was replaced by an IQR approach over LAI. This version is thereafter referred to as the full-range dataset. We built LAI-VI relationships for both datasets separately.

### 2.2. Remotely Sensed Data

We used both Landsat TM and ETM+ images to generate VIs for the in situ LAI observations. Both sensors share the same band designations and spatial/temporal resolutions, thus we did not address between-sensor variability [72]. We selected the closest-in-time Landsat image within 15 days from each LAI measurement date. Each image was subjected to three levels of radiometric/atmospheric corrections: (1) at-sensor radiance; (2) Top of Atmosphere (TOA) reflectance, and (3) surface reflectance. These corrections were made using the Landsat Ecosystem Disturbance Adaptive Processing System (LEDAPS) software [73,74]. LEDAPS first converts digital number (DN) values to at-sensor radiance, which is then converted to TOA reflectance based on solar zenith angle, Earth-Sun distance, bandpass, and solar irradiance. Finally, atmospheric correction routines based on 6S radiative transfer algorithm [75] convert at-sensor radiance to surface reflectance. Clouds were masked using Automatic Cloud-cover Assessment (ACCA) algorithm [74,76], which is a part of the LEDAPS processing package.

We selected five VIs that are commonly employed in LAI related studies: the Simple Ratio (SR) [48], Normalized Difference Vegetation Index (NDVI) [49], Enhanced Vegetation Index (EVI) [53,54], EVI2 [57], and Green Chlorophyll Index (CI_Green_) [78] (Table 1). SR and NDVI were selected as two of the earliest and simplest VIs, widely used in remote sensing applications. EVI is representative of many soil-line based VIs, such as SAVI and ARVI. Compared to NDVI, EVI is less sensitive to soil background and atmospheric noise, and less saturated at high LAI values [53,54]. EVI2 is a version of EVI that does not require the blue band to facilitate the use of data from sensors without that capability [57]. Recent studies demonstrated that EVI2 and EVI perform comparably in LAI estimation at local scales [59,79]. Therefore, we aimed to evaluate EVI2 over multiple locations at large scales using our in situ global dataset. CI_Green_ was originally designed to exploit the relationship of canopy chlorophyll content and visible green reflectance [78,80,81]. It has been shown to outperform many other VIs for predicting LAI at field scales [58,67], but has not yet been tested at a global scale. These VIs have been proved to be effective in estimating crop LAI in many previous investigations (Table S2).

### 2.3. Establishment of the Global LAI-VI Relationships

#### 2.3.1. Exploratory Analysis: Symbolic Regression

In the exploratory analysis, we used symbolic regression to establish LAI-VI relationships for each VI (derived from DN, at-sensor radiance, TOA reflectance or surface reflectance) and each crop type or group of crops: maize, soybean, wheat, rice, cotton, pasture, row crops (all except pasture), and all crops. Symbolic regression is a semi-supervised method that searches a space of mathematical expressions to find the simplest relationship that minimizes estimation errors. Unlike traditional regression methods, symbolic regression does not require the mathematical form of the relationship to be defined.

In this study, the symbolic regression of the LAI-VI relationships was performed through the Eureqa^®^ package [82,83]. Eureqa^®^ identifies the optimal functions based on samples of dependent and independent variables, a set of operators (i.e., addition, subtraction, exponential, power, sine, cosine), and an error metric. We used LAI as dependent variable and VI as independent variable, and defined an operator set consisting of addition, subtraction, multiplication, exponential, logarithmic, and power. For simplicity, we excluded division, and selected invertible functions with only one term (i.e., VI) and a maximum of three coefficients. We used mean squared error (MSE) as the error metric, which Eureqa^®^ aimed to minimize during the search. After Eureqa^®^ obtained the functional forms of the relationships, regression coefficients were estimated using Least Absolute Deviation (LAD) regression [84]. LAD regression minimizes the sum of absolute errors and provides a robust estimation more resistant to outliers [85,86]. The relationships established through symbolic regression and LAD regression are thereafter referred to as the best-fit functions. Each function is restricted to a reasonable VI range, which only produces LAI between 0 and 6 m^2^/m^2^ to avoid extrapolation.

The best-fit functions were evaluated using three goodness-of-fit (GOF) metrics: R^2^, root mean squared error (RMSE), and mean absolute error (MAE). GOF metrics were calculated via a split-sample cross validation method which used 75% of the samples for regression and the remaining 25% for testing. We reported the mean values of GOF metrics after 500 iterates of the cross validation. Since most of the best-fit functions are non-linear, R^2^ (calculated as the regression sums of squares divided by total sums of squares) was not used to compare models but rather to describe the percentage of the total variance of LAI explained by the LAI-VI relationships [87]. In addition, we also produced the median and quantiles of absolute residuals and their distributions along the LAI range as additional model evaluation statistics following the CEOS LPV LAI protocol [2].

#### 2.3.2. Refined Models of LAI-EVI and LAI-EVI2 relationships

In order to account for the measurement errors in both LAI and VI data, and solve the issue of non-constant residual variance found in many of the best-fit functions, we adopted a rigorous statistical method to construct refined models for LAI-EVI and LAI-EVI2 relationships, as EVI and EVI2 were more effective than other VIs (see Section 3.2.2). This method was based on simple linear regression and Theil-Sen estimator after transformations of dependent and/or independent variables, as recommended to the remote sensing community by Fernandes and Leblanc [88].

We first applied power transformations over LAI and/or EVI/EVI2 to eliminate non-linearity, non-normality of the error terms, and non-constancy of the error variance. Selection of optimal transformation forms were based on Box-Cox model of power transformations on the response variable (i.e., LAI), and Box-Tidewell model for power-transformations on the predictor variable (i.e., EVI, EVI2) [89]. A score test (Cook-Weisberg test) for non-constant error variance was also used to ensure homoscedasticity in the selected transformations and models [89,90].

We then used Theil-Sen estimator to estimate coefficients in the simple linear regressions. Theil-Sen estimator is a traditional robust regression tool, which estimates the slope of the regression line by choosing the median slope of lines through all pairs of sample data points [91,92]. It is an unbiased estimator of the real regression slope, and is robust to up to 29% of samples being outlier [91]. The refined models for LAI-EVI and LAI-EVI2 relationships (based on only surface reflectance data) were used in the following evaluations and analysis.

### 2.4. Evaluation of the Global LAI-VI Relationships

The temporal mismatch between LAI measurement and satellite overpass and the different methods used in LAI measurement are two potential error sources in the LAI and VI data respectively. To assess the effects of these two potential measurement errors on the LAI-VI relationships, we analyzed the regression residuals of the overall LAI-EVI relationship (including all crop types) and conducted an ANOVA test with Welch’s correction on non-homogeneity of variances. The pairwise comparisons were accomplished using Dunnett's Modified Tukey-Kramer pairwise multiple comparison test (*α* = 0.05), which is suitable for unequal sample sizes and has no assumption of equal population variances.

Since we did not have an independent testing set with globally distributed samples, we adopted a site-based evaluation procedure to evaluate the validity of the approach to build global LAI-VI relationships, and assess the universality of these relationships. In this analysis, we used four crop types with large sample size: maize, soybean, wheat, and pasture. For each crop type, we used data only from sites with at least 10 samples. Then for each crop and each site, we first fitted a model using the method described in Section 2.3.2 and data from all other sites, and then calculated GOF metrics of the model using data for the site of interest. We compared coefficients and GOF metrics of models constructed using different sets of data. This analysis reveals the universality of the LAI-VI relationships as well as the leverage each individual site has over the global relationships.

### 2.5. Preliminary Validation and Example Applications at Three Spatial Scales

We applied the LAI-VI relationships to remote sensing data at three different spatial resolutions/scales. This served as a preliminary validation effort and as examples of potential applications of our LAI-VI relationships for readers who may be interested in applying the relationships in their own studies. Note that in this analysis, the reference LAI data, albeit modeled in nature, were treated as reliable sources of LAI estimates with credible scientific basis as opposed to in situ measurements that would provide direct evidence of the error and bias in our estimates.

#### 2.5.1. Field Scale Application

We measured LAI of maize canopy weekly and obtained high spatial resolution imagery (0.8m) from an airborne sensor in two maize fields located in northwest of Deforest Wisconsin (43.27° N, 89.40° W), US. This site and LAI monitoring efforts are described in detail in [64,93].

During the experiment, multispectral images were collected using a 6-sensor Tetracam Multi Camera Array (MCA) system (Tetracam Inc., Chatsworth, CA, USA) mounted on the underside of a Cessna 3-passenger airplane. MCA sensors were centered at 450, 570, 620, 650, 670, and 860 nm with a uniformly-averaged 10 nm band width. Images were collected on four dates during the 2012 growing season (5/25, 6/22, 7/30, 8/29) and eight dates during the 2013 growing season (6/4, 7/2, 7/24, 8/1, 8/13, 8/20, 9/5, 9/23) from ~1200 m (~4000 ft) above the ground surface, leading to a ground instantaneous field of view of (GIFOV) of ~0.8 m. Each sensor produced a separate image; individual images were co-registered using Tetracam’s Pixelwrench software and georeferenced in ArcGIS 10 (ESRI, Redlands CA). To convert MCA images from DN to surface reflectance, we used four control points at the study site which were present in each image: surface water, tarmac road, concrete parking area, and healthy green grass. At least nine spectral reflectance measurements at 1 nm resolution were collected for each of these control points using an ASD handheld spectrometer (Analytical Spectral Devices, Inc., Boulder, CO, USA). These measurements were then averaged at 10 nm wavelengths intervals corresponding to each of the six MCA sensors. Spectrometer-derived mean surface reflectance was linearly regressed to MCA-derived DN values to produce a DN-surface reflectance relationship for each image. Two images (6/22/2012 and 6/4/2013) were discarded due to poor fits between MCA and spectrometer data (*R*^2^ <0.60). The retained images had a mean *R*^2^ of 0.72. The relationships developed using linear regression were applied to convert the image DN values to surface reflectance, which were then used to compute VIs.

The LAI measurements were made using a Li-Cor LAI-2200 (Li-Cor Biosciences, Lincoln NE) plant canopy analyzer at approximately weekly intervals throughout the 2012 and 2013 growing seasons. LAI measurements were taken as the average of 20 below- and 20 above-canopy readings, and were collected under diffuse light conditions (sunrise, sunset, or full cloud cover). LAI measurements were collected the same day as MCA image collection when possible; when LAI and MCA images were not collected on the same day, LAI values were linearly interpolated between measurement dates to estimate the LAI at the time of image collection.

Finally, local relationships between field measured LAI and each VI were established following the same processes described in Section 2.3.2, and used to produce LAI maps for comparison with maps produced using the global LAI-VI relationships.

#### 2.5.2. Local Scale Application

The second validation process was conducted using Landsat imagery acquired in the Central Valley of California. The study area features one Landsat footprint, which has a heterogeneous agricultural landscape with various crop types. The reference dataset was a LAI map obtained from the Provisional Landsat LAI Products developed by the NASA Earth Exchange (NEX) program [39]. It was produced from a Landsat ETM+ image acquired in July 2005 using the radiative transfer algorithm adapted from the MODIS LAI product. We produced a LAI map using the global LAI-VI relationship and the same Landsat ETM+ surface reflectance image in the NEX product. We chose to use the global overall relationships only (i.e., not crop-specific) as no crop-type map was available in this area.

#### 2.5.3. Regional Scale Application

The third application was implemented at 1km resolution using MODIS data within the northwestern corner of the state of Iowa (USA), a region spanning 15 counties. This area is primarily comprised of maize and soybean fields. A crop map of this region for 2009 was extracted from the Crop Data Layer (CDL), a crop type dataset derived from AWiFS data at 56m spatial resolution (http://nassgeodata.gmu.edu/CropScape/) [94]. To be consistent with the MODIS images, the CDL map was aggregated to 1 km resolution using a square-wave filter, and the resulting map shows maize and soybean cultivated area fractions for each 1 km pixel.

We used the MODIS Collection 5 Nadir BRDF-adjusted reflectance (NBAR) product (MCD43A4) [95] to produce the LAI maps using the global LAI-VI relationships. The NBAR data were produced as 8-day composite at 500m spatial resolution, but were aggregated to 1km resolution and a 16-day interval before LAI processing. Based on MODIS reflectance and the crop map, two sets of LAI maps were produced: one based on the global overall LAI-VI relationship (i.e., not crop-specific), and the other based on global LAI-VI relationships for maize and soybean. In the latter maps, LAI was computed as a weighted average of maize and soybean LAI based on the fractions determined from the crop map.

These maps were then compared to the reprocessed MODIS Collection 5 LAI products by the Beijing Normal University Land-Atmosphere Interaction Research Group [96]. This LAI product is an improved version of the original MODIS LAI product [97], which overcomes the issues of data noise and gaps by applying a modified temporal/spatial filter. It has a 1km spatial and 8-day temporal resolution, but was aggregated to 16-day. Besides comparing the LAI maps, we also constructed and compared growing season LAI time-series for the two dominant crops in Iowa—maize and soybean—using both global LAI-VI relationships and MODIS BNU LAI products. Since MODIS has a coarse resolution of 1 km, which is larger than most of the soybean and maize fields, the LAI time series used average values of only the pure maize or soybean pixels, which are defined as pixels with more than 90% areas occupied by each crop.

## 3. Results

### 3.1. Description of the In-Situ LAI Data Set

**Figure 1:**
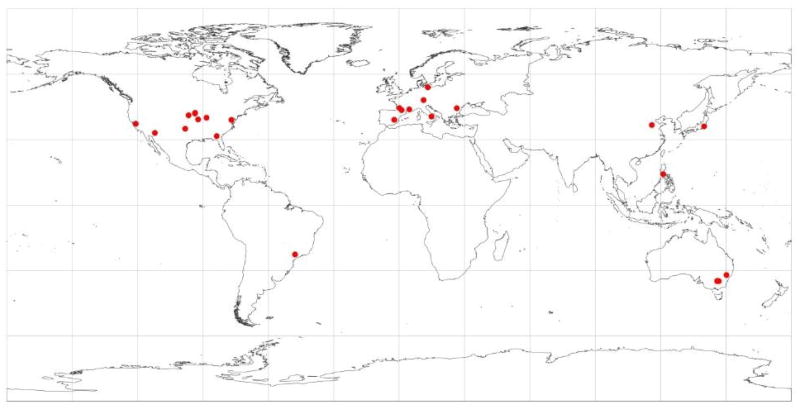
Location of in situ leaf area index (LAI) measurement sites.

The initial dataset contained 2,086 unique records of LAI from 31 sites located in 11 countries (Figure 1; Table S1). A data record refers to a single observation of LAI or the average of all observations within a Landsat pixel (30 by 30 m) on a given date. Each record is associated with VIs derived from the Landsat pixel as well as crop type, measurement method, date, and other ancillary information. The LAI were measured mainly between 2000 and 2012.

In the quality control process, 209 samples were removed according to rule one, 328 for rule two, 50 for rule three, and another 40 for rule four (Table S1). Of the 40 outliers in rule four, 21 were maize, 14 for wheat, 6 for rice, and 9 for pasture. There were no outliers for soybean and cotton. In rule two, when each site was examined, we identified three sites (Agro, SMAPEx2, and SMEX02-WC) with apparent abnormalities in the data. The Agro site was excluded because over 1/3 of maize measurements had unrealistically high LAI values (>8 m^2^/m^2^). The SMAPEx2 site was excluded because ancillary data showed that many of the LAI measurements were taken after crop maturity and contained a mix of brown and green LAI, while this study focuses only on green LAI. Finally, the SMEX02-WC site was excluded due to unreasonably high values of CI_Green_ compared to literature reported values as well as the rest of the dataset [58,67,78,80,81]. Detailed information regarding these three sites can be found in Appendix B.

Our final quality-controlled dataset consists of a total of 1,459 LAI records from 15 different crop types, including maize, soybean, wheat, rice, cotton, pasture, tuber crops, vegetables, barley, canola, and sunflower. Six major crops (i.e., maize, soybean, wheat, rice, cotton, and pasture) occupied 77% of the dataset (Table 2). The final dataset was drawn from five continents, but most of the observations came from the US, Europe, and Australia (Table 2). Due to limited LAI measurements and frequent clouds in tropical areas, there is only one site in South America and no data from either Africa or South Asia, which explains the relatively small sample size for rice.

**Figure 2:**
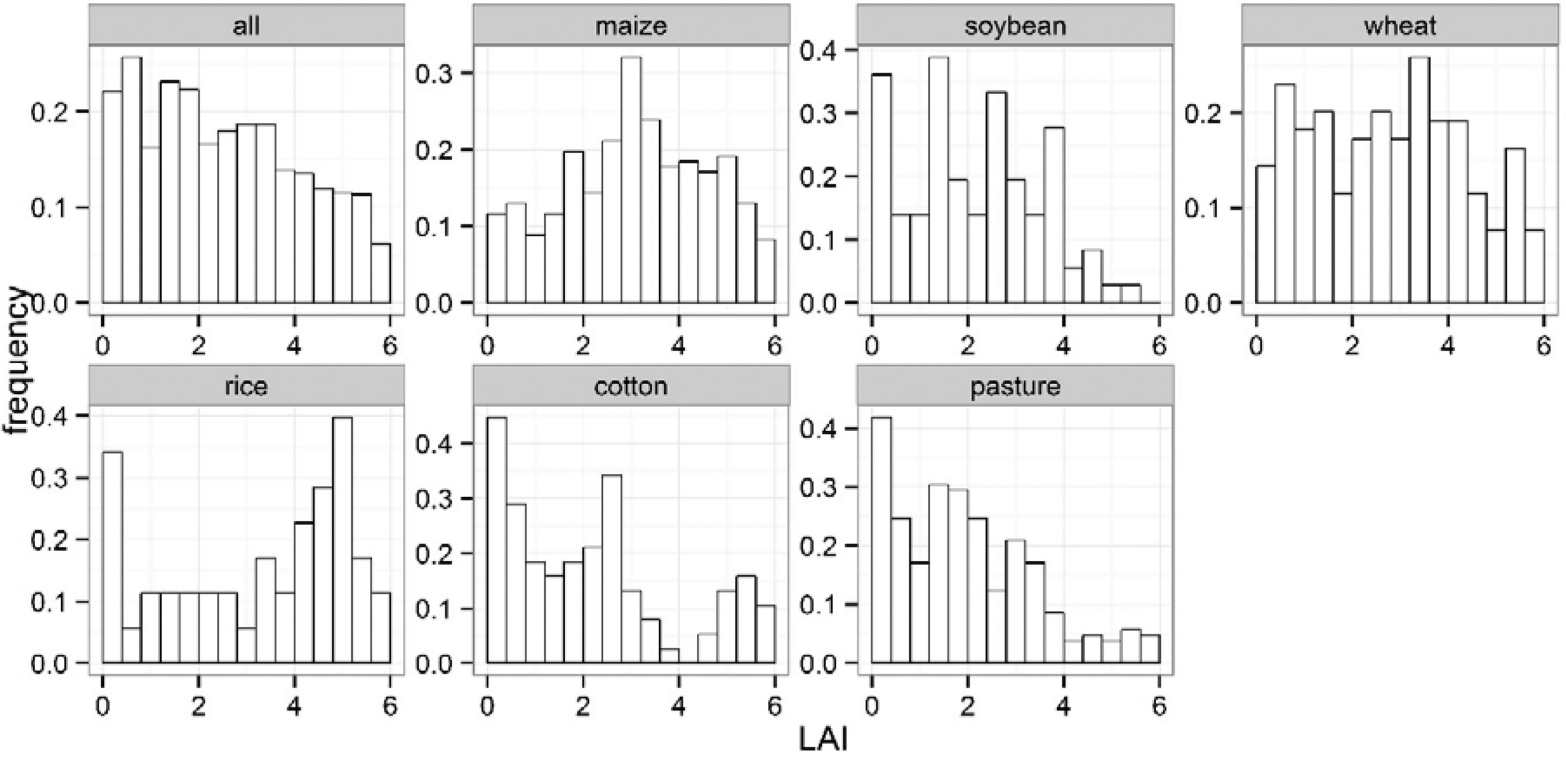
Frequency distribution of the global LAI dataset organised by crop types.

**Figure 3:**
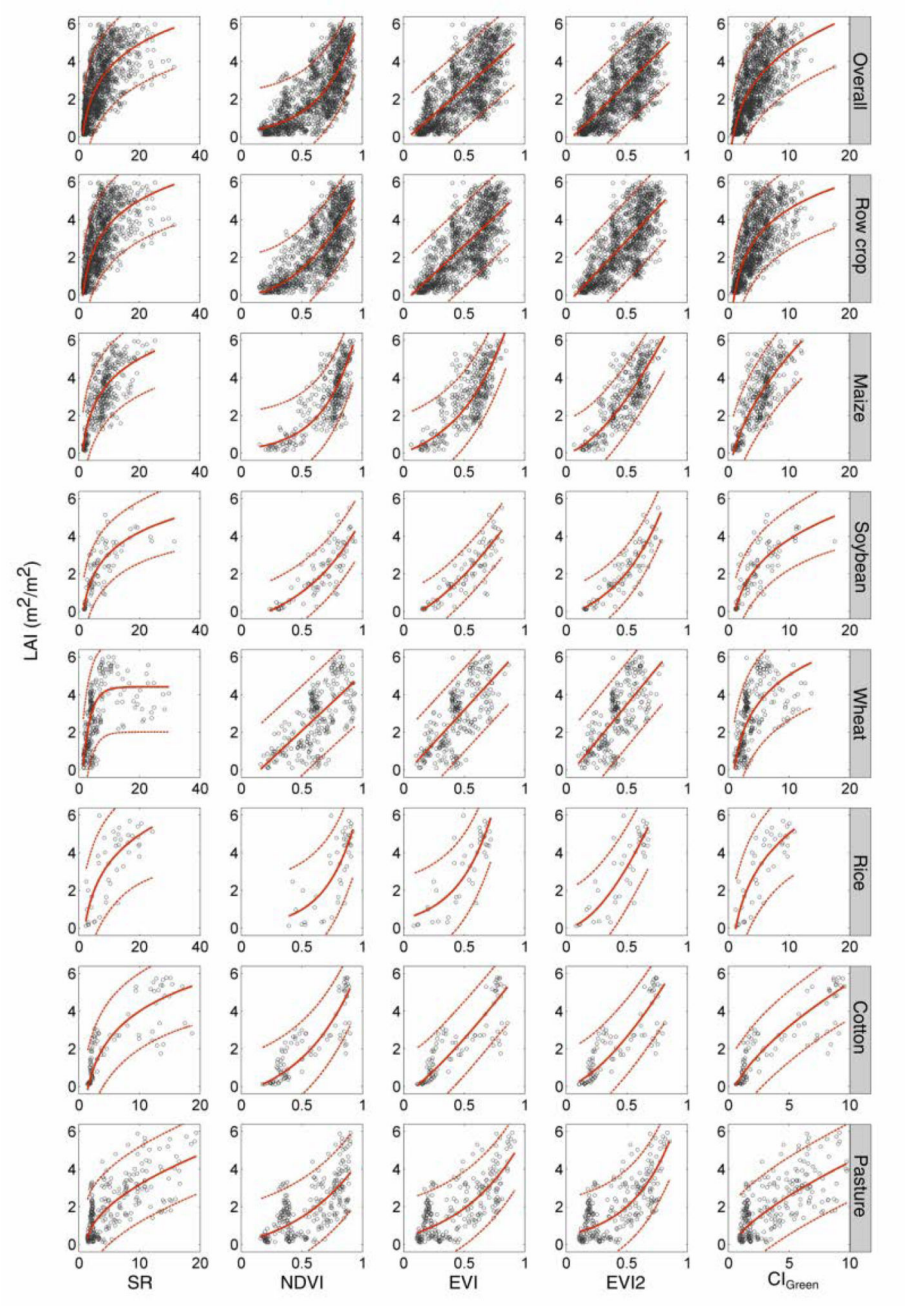
Best-fit functions of global LAI-VI relationships based on in situ LAI and surface reflectance based VIs (SR, NDVI, EVI, EVI2, and CIGreen) for the overall dataset as well as different crop types. Best-fit functions are plotted by solid red lines, and prediction intervals (95%) are presented in dashed red lines. Note that all the curves are fitted in the form of *LAI* = *f*(*VI*), although in this figure, LAI is plotted on the X axis instead of the Y axis to better address the saturation issue of the normalized VIs (NDVI, EVI, and EVI2).

The distribution of LAI values in our dataset is positively skewed with more samples in the lower range than the upper end (Figure 3). Each crop type has a full range of LAI values (from less than 0.12 to more than 5.5 m^2^/m^2^) which nevertheless distribute differently (Table 2; Figure 2). The distribution of maize is approximately unimodal with a peak in the middle (~3 m^2^/m^2^), and the distribution for pasture is positively skewed. For soybean, wheat, rice, and cotton, the distribution is not as clear or in some cases multimodal.

In the dataset, the in situ LAI measurement method include (1) destructive harvesting and direct determination of one-sided leaf area; (2) indirect optical method using the LAI2000 (Li-Cor, Lincoln, NE, USA) or AccuPAR (Decagon Devices Inc., Pullman, WA, USA) instruments; and (3) indirect optical method through analysis of Digital Hemispherical Photography (DHP), which approximates canopy gap fraction from analysis of digital images obtained with a fish-eye lens. Overall, 73% of the LAI observations were collected using the LAI2000 or AccuPAR, 16% were from direct harvesting, and 11% were acquired with hemispheric photography, all of which were located in Europe (Table 2). Within each measurement method, there is a full range of LAI with similar means and variance.

The full-range dataset results in 1784 in situ LAI records ranging from 0.002 to 8.45 m^2^/m^2^ (Table S3). The maximum LAI values for maize, wheat, and rice are up to 7.8 to 8.5 m^2^/m^2^, while for soybean, cotton, and pasture, the peak LAI are below 7 m^2^/m^2^. Note that, unlike in the other version, we included data from Agro site in the full-range dataset, as most of the LAI values above 7 m^2^/m^2^ were found in Agro.

### 3.2. Exploratory Analysis of the LAI-VI Relationships

#### 3.2.1. Form and Shape of the Best-Fit-Functions

The best-fit-functions, constructed based on symbolic regression and LAD regression, allow for initial assessment the LAI-VI relationships for each crop type and VI. We found that the best-fit functions are all statistically significant, indicating that there is a monotonically increasing relationship between LAI and VI at a global scale (Figure 3), even with the presence of confounding variables and measurement errors. All the best-fit functions have simple mathematical forms (i.e., linear, power, exponential, or logarithm) with two or three coefficients, which allow easy applications. A complete list of all best-fit-functions, coefficient confidence intervals, and GOF metrics can be found in Tables S2 and S3.

Generally, the majority of the LAI-VI relationships are non-linear. Relationships established using ratio based VIs (i.e., SR and CI_Green_) are concave curves bending towards the X (LAI) axis and described by logarithmic and power-type functions. In contrast, relationships established using normalized VIs (i.e., NDVI, EVI, and EVI2) exhibit convex curves bending towards the Y (VI) axis, typically with an exponential form (in some cases power), indicating saturation at high values of LAI. Saturation is less common in relationships using EVI and EVI2 compared to NDVI, and in some cases EVI/EVI2 are linearly related to LAI, indicating an ability to resolve LAI differences over a wider range of canopy conditions.

Both the mathematical form and the coefficient values of LAI-VI relationships change with crop types (Figure 3; Table S2). For example, for NDVI, the best-fit function for wheat is linear while for all other crops the relationship is exponential. Soybean, cotton, and pasture exhibit the same form of equation, but their coefficients are substantially different. This leads to differing patterns of saturation (or a horizontal asymptote in Figure 3 indicating an inability to differentiate between high values of LAI) across crop types for NDVI. For instance, NDVI saturates at LAI 3 m^2^/m^2^ for maize and rice, at 4 m^2^/m^2^ for pasture, and 5 m^2^/m^2^ for cotton, while for soybean and wheat, the relationships are close to a straight line.

#### 3.2.2. GOF Metrics of the Best-Fit-Functions

Overall, the surface reflectance-based VIs explain more than 50% of the variance in observed LAI (inferred from *R*^2^ values), with RMSE between 0.7–1.2 m^2^/m^2^ and MAE between 0.5–1.05 m^2^/m^2^ (Table S3). The median absolute residuals are between 0.3–0.9 m^2^/m^2^, and 95% of the absolute residuals are below 2.5 m^2^/m^2^.

The goodness-of-fit varies across crop types and the choice of VI. The difference is greater between crop types than between VIs for the same crop (able A3), indicating that available ancillary data regarding land cover should be used when applying a universal relationship at a local scale. For overall and row crop relationships, the RMSE and MAE are ~1.1 m^2^/m^2^ and ~0.88 m^2^/m^2^ respectively. In the six major crops, soybean has the lowest RMSE (~0.65 m^2^/m^2^) and MAE (~0.56 m^2^/m^2^), followed by cotton (RMSE ~ 1 m^2^/m^2^ and MAE ~ 0.74 m^2^/m^2^). Maize and pasture have an intermediate range of RMSE (~1 m^2^/m^2^) and MAE (~0.8 m^2^/m^2^). The goodness-of-fit for wheat and rice are weaker, with RMSE at ~1.3 m^2^/m^2^ and MAE ~1 m^2^/m^2^. Within each crop type, there is a relatively small difference in GOF between VIs. For crop-specific relationships, both EVI and EVI2 result in the lowest RMSE and MAE for all crops except for cotton. For overall and row crop relationships, the difference in GOF between VIs is negligible (<0.03 m^2^/m^2^ RMSE and MAE). In general, SR performed the worst, NDVI and CI_Green_ performed similarly, and EVI and EVI2 performed the best. We note that EVI2, which does not rely on the blue band to minimize the atmospheric effects, provides a comparable relationship to EVI for most crops.

#### 3.2.3. The Effect of Levels of Radiometric/Atmospheric Corrections

**Figure 4:**
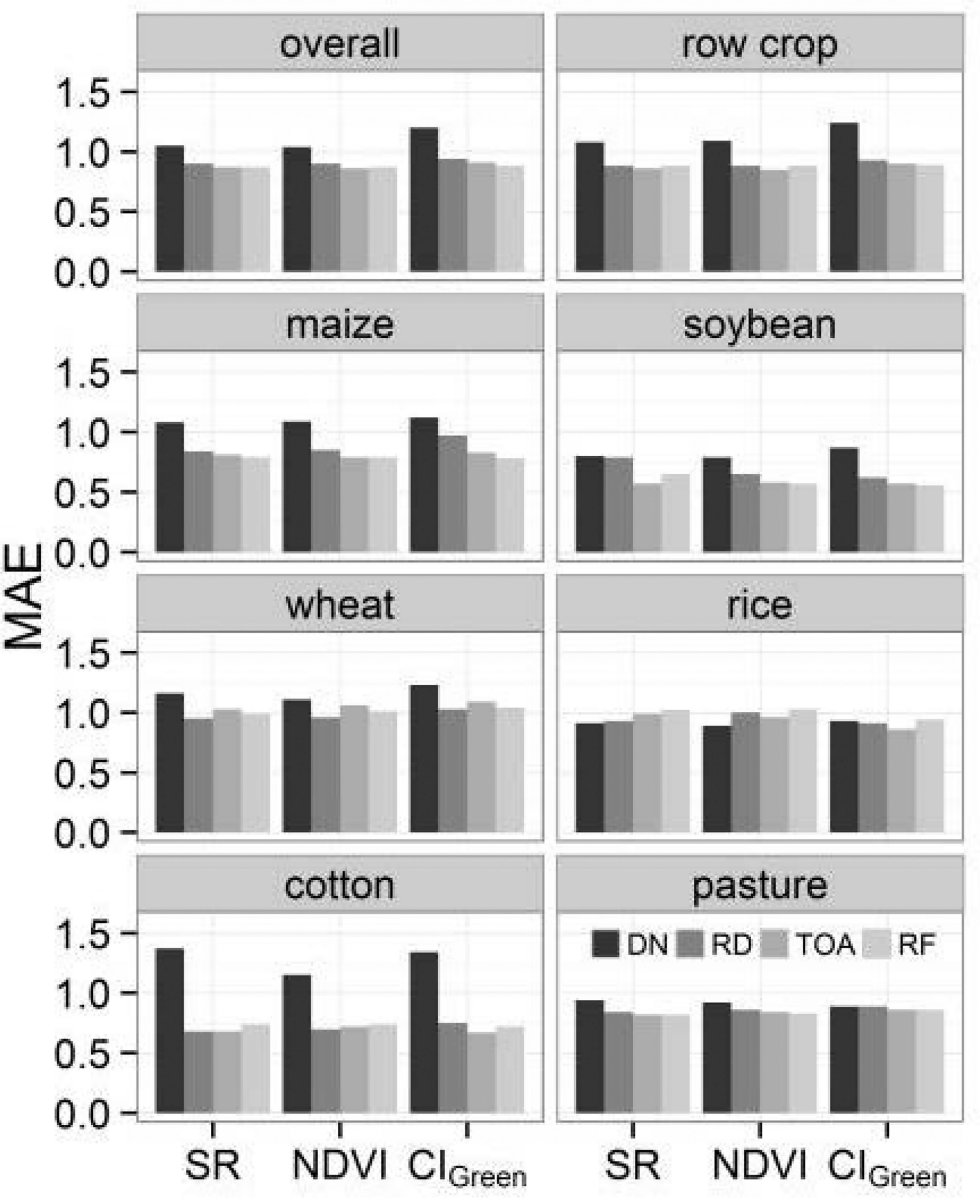
MAE (cross validation) of the best-fit functions for different crops across levels of radiometric/atmospheric corrections. In the legend, RD presents for radiance, TOA is the TOA reflectance, and RF is the surface reflectance. Note that EVI and EVI2 are not included in this figure, since these VIs can only be calculated from reflectance data.

The radiometric/atmospheric corrections have significant effect on the GOF of LAI-VI relationships (Figure 4). For all crop types except rice and pasture, ANOVA test shows that the absolute errors are significantly different (*p* <0.001) among relationships fitted using different radiometrically corrected remote sensing data for all VIs. The errors of DN based relationships are significantly greater than those of any other level of data based on the pair wise comparison. In many crops (i.e., overall, row crop, maize, soybean, pasture), there is a decreasing trend in the MAE from radiance, TOA reflectance to surface reflectance, but the differences are not statistically significant (except for maize). For some crops, (e.g., wheat, rice, cotton), it is interesting to note that the error from radiance could be the lowest, while that of surface reflectance is the highest, but these differences are also not statistically significant.

In sum, we found that any form of reflectance conversion is preferred over DN when computing VIs, as the inclusion of sun-sensor geometry normalizes the illumination and viewing conditions and provides physically meaningful observations that are comparable across sensors, times, and locations. Since the best-fit-functions are affected by many factors beside the radiometric corrections on remote sensing data, it is difficult to draw any general conclusions with regard to the difference among radiance, TOA reflectance, and surface reflectance.

#### 3.2.4. The Best-Fit Functions Based on Full-Range Dataset

We also established best-fit-functions for each VI (only reflectance based) and each crop using the full-range dataset (Tables S6 and S7). The relationship curves resemble those fitted based on the truncated dataset (Figure 3, Figure S6), while function forms and coefficient values are slightly different (Tables S5 and S6). In Figure S6, for the overall, row crop, maize, and rice relationships, most of the above 6 m^2^/m^2^ LAI values are always outside the confidence interval (dashed red line), and the saturation issue is apparent in the NDVI, EVI, and EVI2 plots when LAI is above 6 m^2^/m^2^. This means that VIs derived from reflectance are not sensitive to LAI when the canopy is very dense (LAI >6 m^2^/m^2^). Note that these findings are not entirely applicable to wheat (Figure S6), where the additional samples ranging from 6 to 8.5 m^2^/m^2^ has expanded the relationship’s prediction range, and linear or quasi-linear relationships were concluded for NDVI, EVI, and EVI2.

As for the GOF metrics, the additional samples in the full-range dataset have increased the RMSE by 0.2–0.3 and MAE by 0.1–0.2 compared to the truncated dataset, as the LAI-VI relationships are unable to provide reasonable predictions for large LAI values in the majority cases.

### 3.3. LAI-EVI and LAI-EVI2 Relationships Based on Theil-Sen Regression

From the diagnostic plots of the best-fit-functions established through symbolic regression, we found that the residuals of some relationships show non-constant variances and/or non-linearity patterns rendering the regression model inefficient (Figure S8). This is partly attributed to the presence of errors in both LAI and VI observations. Therefore, we constructed rigorous statistical models of the LAI-VI relationships that conform to the i.i.d (independent, identical distributed variable) assumption. Since the relationships of EVI and EVI2 are the strongest, especially for crop-specific relationships, and have reduced saturation problems compared to NDVI (Figure 4), we used only EVI and EVI2 to establish the refined models for LAI estimation using Theil-Sen estimator.

**Figure 5:**
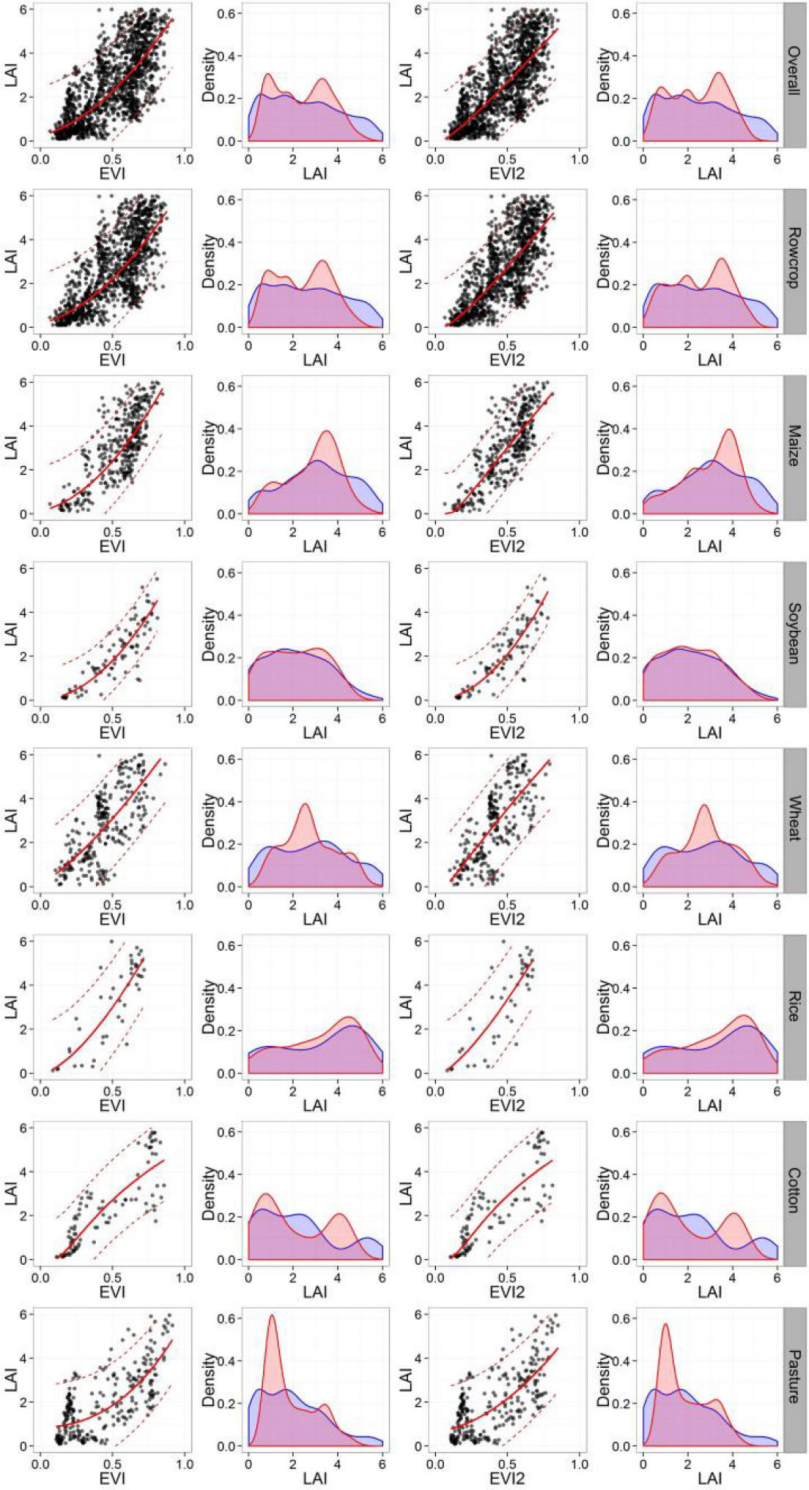
LAI-EVI and LAI-EVI2 relationships based on Theil-Sen regression and the density distributions of the measured and predicted LAI. The first and third columns show scatter plots between LAI and EVI/EVI2 as well as the relationship (solid red line) and prediction interval (dashed red line) based on Theil-Sen regression. The second and fourth columns show density distributions of the measured (blue) and predicted (red) LAI based on EVI and EVI2 respectively.

The LAI-EVI and LAI-EVI2 relationships based on Theil-Sen regression are all statistically significant and conform to the assumption of independent and identical distribution of errors and homoscedasticity of residuals, according to Box-Cox and Box-Tidewell Cook transformation as well as the Weisberg test (Figure 5). The relationships have a RMSE around 0.7–1.1 m^2^/m^2^ and MAE between 0.5–0.9 m^2^/m^2^ (Table 3). Variability of GOF metrics is in accord with results from exploratory analysis, where the overall, rowcrop, and wheat relationships give higher RMSE, and the soybean relationship has the lowest error. The GOF metrics also suggest that EVI2 performs comparably to or better than EVI in the prediction power across all crops (Table 3), proving that EVI2 can be used as a robust estimator of LAI when data from the blue band is not available.

Density distributions of predicted LAI generally match with those of measured LAI, especially for soybean and rice, though some discrepancies exist (Figure 5). For example, the predicted distributions capture multi-modal behavior well, but tend to exaggerate the amplitude of the modes (i.e., maize, wheat, pasture). For cotton, the peaks and valleys of the predicted prediction do not match the measured distribution, potentially due to the unbalanced sample. In addition, we again observe no significant difference in the performance between EVI and EVI2.

We also provided LAI-EVI and LAI-EVI2 relationships based on Theil-Sen regression for the full-range dataset (Table S8, Figure S7), and similar conclusions can be drawn as the best-fit-functions. Again, we found that EVI and EVI2 can only provide reasonable estimations for the entire range of LAI values for wheat, while for overall, row crop, and maize, EVI and EVI2 saturate when LAI is above 6 m^2^/m^2^. As a result, for the following analysis and assessment, we only used the relationships based on truncated dataset, and readers are advised to be cautious when interpreting the result to avoid extrapolation.

### 3.4. Evaluation of the LAI-EVI/EVI2 Relationships

#### 3.4.1 Analysis of the Errors from Temporal Mismatch and Measurement Methods

**Figure 6:**
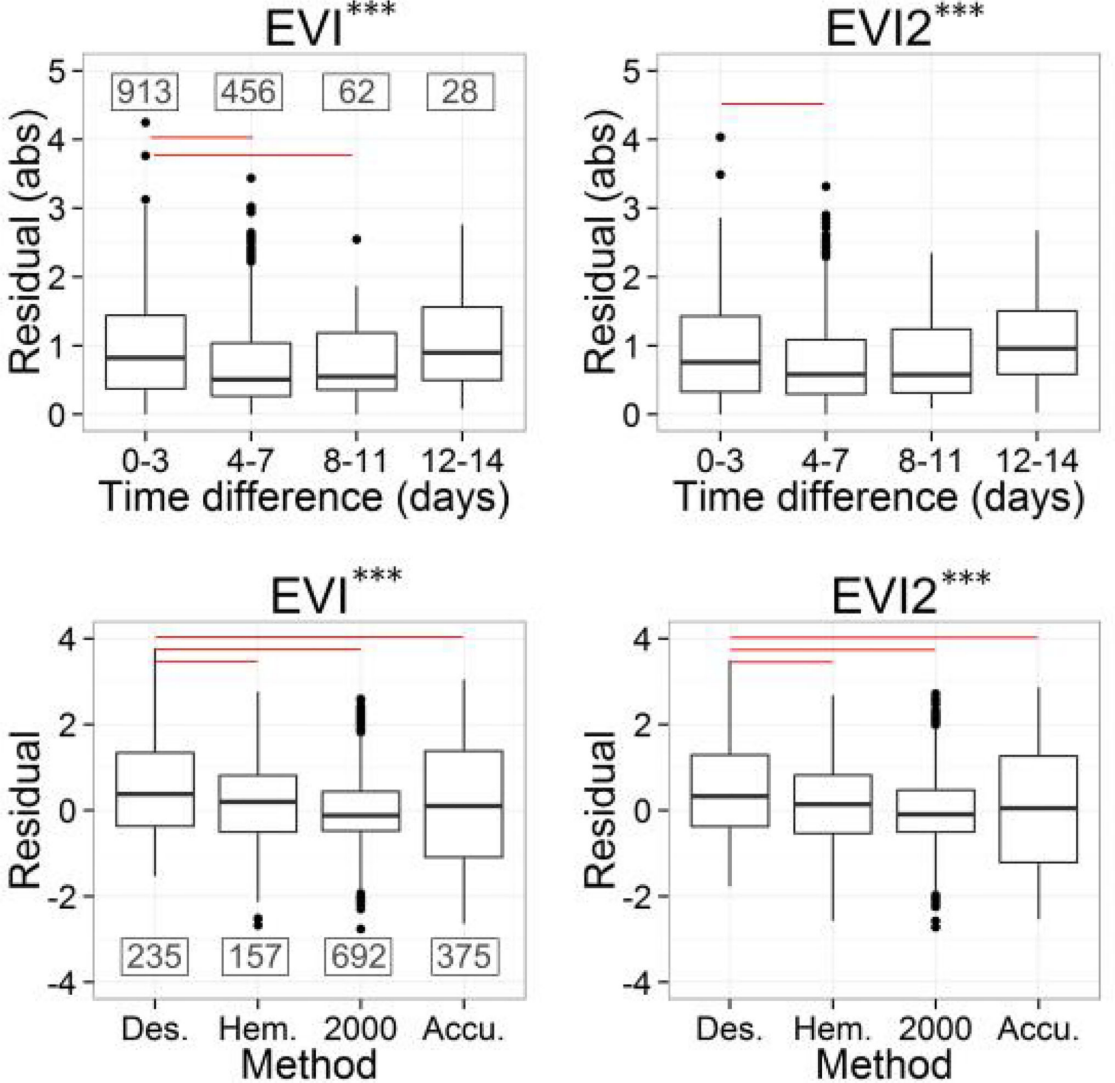
Boxplots of the absolute residuals summarized by temporal mismatch between LAI observations and satellite image acquisition (first row) and residuals summarized by measurement methods (second row) for the LAI-EVI and LAI-EVI2 relationships. The numbers in the EVI panel are the sample sizes for each category. In the boxplot, two ends of the boxes correspond to the first and third quartiles of the data, and the whiskers extend from the edges to the highest/lowest value that is within 1.5 * IQR of the hinge, where IQR is the inter-quartile range. Data beyond the end of the whiskers are plotted as points. The significance levels from ANOVA test (with Welch’s correction on non-homogeneity of variances) are shown on the title of each panel. The significance codes represent: *** <0.001; ** <0.01; * <0.05. Significantly different pairs (*α* = 0.05) identified by the pairwise comparisons based on Dunnett's Modified Tukey-Kramer test are connected by lines. The red line indicates that the mean of the left group is significantly larger than that of the right group.

Overall, we found a significant effect of temporal mismatch on the LAI-EVI and LAI-EVI2 relationships based on results from the ANOVA test (Figure 6). The residuals for temporal mismatch between 12–14 days are the greatest of all, although this is not statistically significant based on results of the Tukey-Kramer test probably due to the unequal sample sizes. The residuals of the 0–3 day group are statistically greater than that of the 4–7 days group for both EVI and EVI2, likely due to the fact that the majority of samples fall in the 0–3 day group. Although sample sizes are much smaller for larger temporal mismatch, there is still an increasing trend of the residuals starting from 4-day temporal mismatch.

According to the ANOVA test, there is also an overall effect of LAI measurement methods on the LAI-EVI and LAI-EVI2 relationships (Figure 6). Residuals from destructive sampling are significantly greater than those from the optical methods, while there is no significant difference among the optical methods. These findings support previously reported underestimation issues associated with optical methods, which is mainly due to the assumption of randomly distributed foliage elements within the canopy [14,17,98,99]. In fact, measurements of non-contact optical instruments made at a single angle correspond to effective LAI, which can be corrected to true LAI based on the ratio of non-foliage area to plant total area and foliage clumping index [126,127]. Nevertheless, such corrections are complicated and usually made at each site based on site-specific conditions. The result here implies the potential uncertainties in the global LAI-VI relationships brought by the inconsistency of measurement methods and data correction procedures.

#### 3.4.2. Site-Based Evaluation on Global Universality

**Figure 7:**
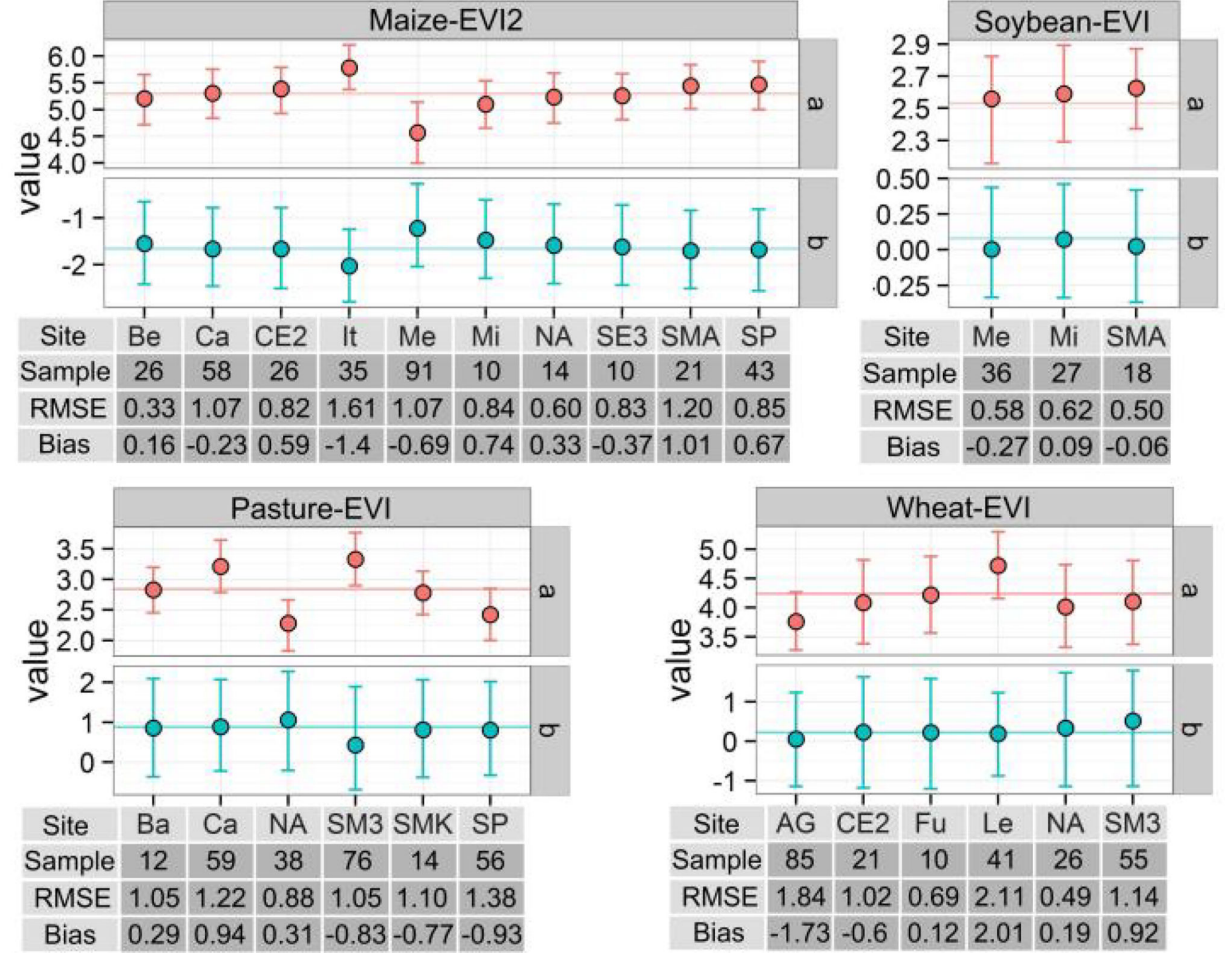
The variation of coefficient values from site-based validation for crop types with sufficient sample size: maize, soybean, wheat, and pasture. For each crop, the best model from Table 3 was used based on RMSE. Each site containing adequate samples (at least 10 samples) for a crop type is used once as a test site, while the rest sites serve in regression to obtain coefficient values. Coefficient values are plotted with filled circles corresponding to the test site displayed on the X axis. The horizontal line shows the value of coefficients (data from Table 3). The sample sizes, RMSE, and bias of each testing site are displayed in tables under X axis. The short names for each site are used on X axis: AG: AGRISAR, Ba: Barrax, Be: Beltsville, Ca: California, CE2: CEFLES2, Fu: Fundulea, It: Italy, Le: Les Alpilles 1, Me: Mead, Mi: Missour, NA: NAFE06, SE3: SEN3EXP2009, SM3: SMAPEx3, SMA: SMEX02-IA, SMK: SMEX03-OK, SP: SPARC.

The site-based evaluation result suggests that variation of coefficient values are minor among models fitted with difference subsets of samples selected by leave-one-site-out method, as most of the coefficient values are close to and distributed around the coefficient fitted using all samples (Figure 7). This is expected, as the global LAI-VI relationships reflect an average condition of the constituent sites. For some sites (i.e., Italy and Mead (maize); California, NAFE06, SMAPEx3, and SPARC (pasture); and AGRISAR and Les Alpilles 1 (wheat)), the corresponding coefficients fitted using samples from other sites are far from the global value as well as the rest of the leave-one-site-out models. These sites tend to have the largest sample sizes, and therefore this difference reflects a large leverage on the global relationships.

The RMSE, bias, and mean absolute percentage error (MAPE) values from leave-one-site-out evaluation indicate how well models fitted with one set of pooled samples can be applied to an independent site outside the training samples. Most RMSE values are <1.2 m^2^/m^2^, with more than half <1 m^2^/m^2^, bias values are between ± 1 m^2^/m^2^, and often ± 0.5 m^2^/m^2^ (Figure 7). The MAPE is between 10% and 60%, with 2/3 of the sites below 40%. Since the LAI measures are relatively low (<1 m^2^/m^2^) in a lot of sites, the MAPE values tend to be high. Only three crop-sites (maize-Italy, wheat-AGRISAR, and wheat-Les Alpilles 1) have biases >1.5 m^2^/m^2^. Performance tends to be the worst for wheat, which may be partly explained by the fact that wheat is the most geographically diverse crop in our sample. The scatter plot in Figure 4 and Figure 6 both confirm this point.

Across all crops, the small variation in coefficient values and relatively low RMSE values observed in Figure 7 support the universality of the relationships and suggest that global LAI-VI relationships can be applied to independent datasets with a certain degree of confidence. However, there is a non-zero bias for each crop-site combination, albeit a small one in most cases. These biases could be positive or negative with varied values centered on zero, which reflects the random error term in the regression model of the global LAI-VI relationships; such error is inherent in the “one place, one time, one equation” issue as a result of the diverse nature of local LAI-VI relationships.

**Figure 8:**
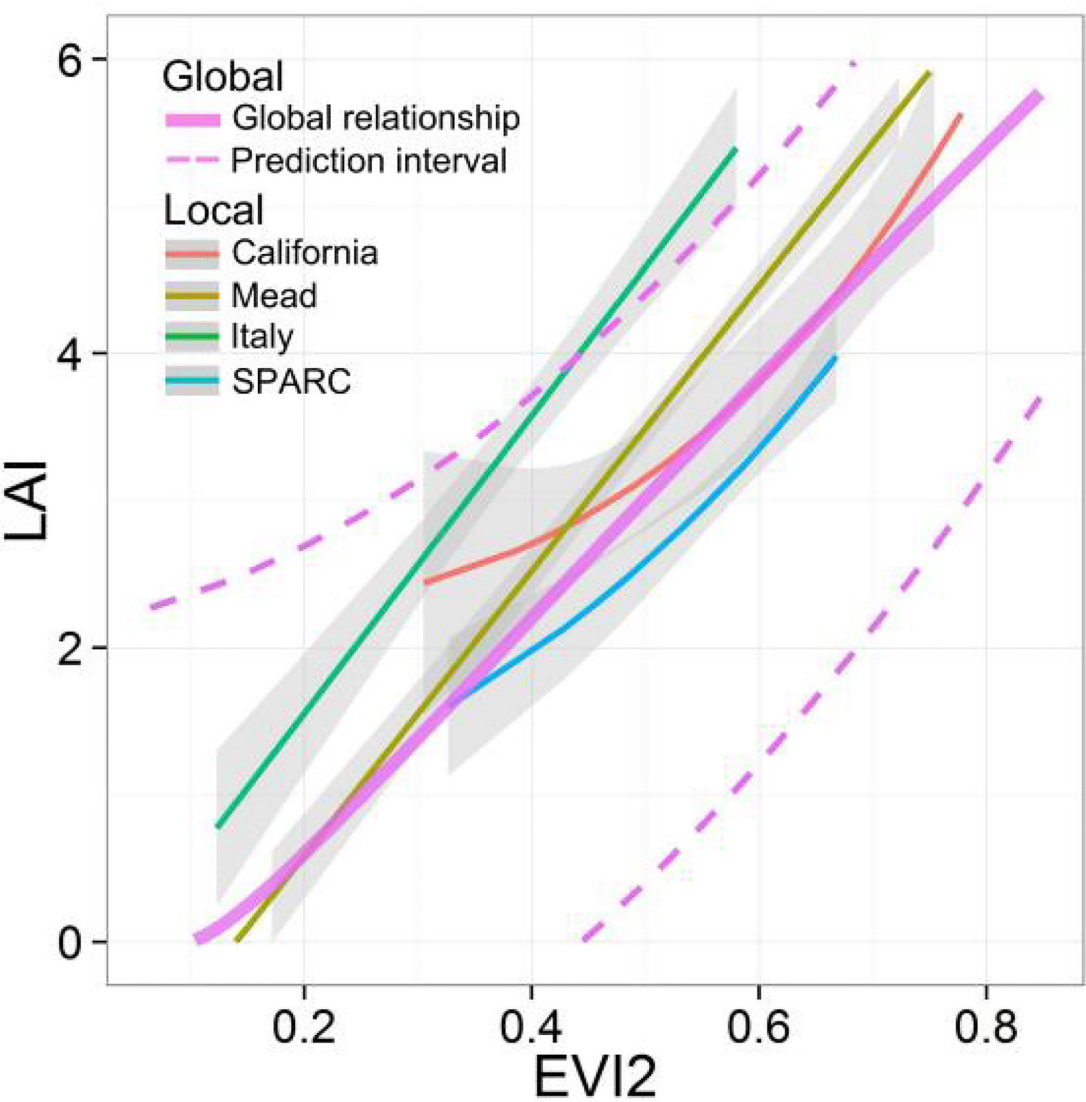
Local LAI-EVI2 relationships of maize for four major sites compared to the global maize relationship. The thin colored lines are the best-fit functions for each site. The thick solid rose-colored line refers to the global relationship, with dashed rose line being the prediction interval. Gray shaded areas are the 95% confidence intervals.

Figure 8 visualizes the one place, one time, one equation issue in a more direct manner. In this figure, we compared local LAI-EVI2 relationships of maize in four sites (with sample size greater than 30) as well as the global LAI-EVI2 model (Table 3). The trend of site-specific relationships are similar to the global relationship, but each relationship has a unique shape and location in the LAI-EVI2 space, leading to bias when using one curve in a different location. Taking the Italy site as an example, the global and any other local relationships function would result in an underestimation of LAI, although the local relationship itself is very strong (*R*^2^ = 0.85). This also explains the large RMSE for the Italy site in Figure 8. This phenomenon is the source of bias when applying a global LAI-VI relationship to a local site, or applying a local relationship from one place to another location.

### 3.5. Preliminary Validation and Example Applications

To further explore the applicability of global relationships to local sites, we conducted three comparisons between estimations of global relationship and independent datasets at field to regional scales outlined in Section 2.5.

#### 3.5.1. Field Scale

**Figure 9:**
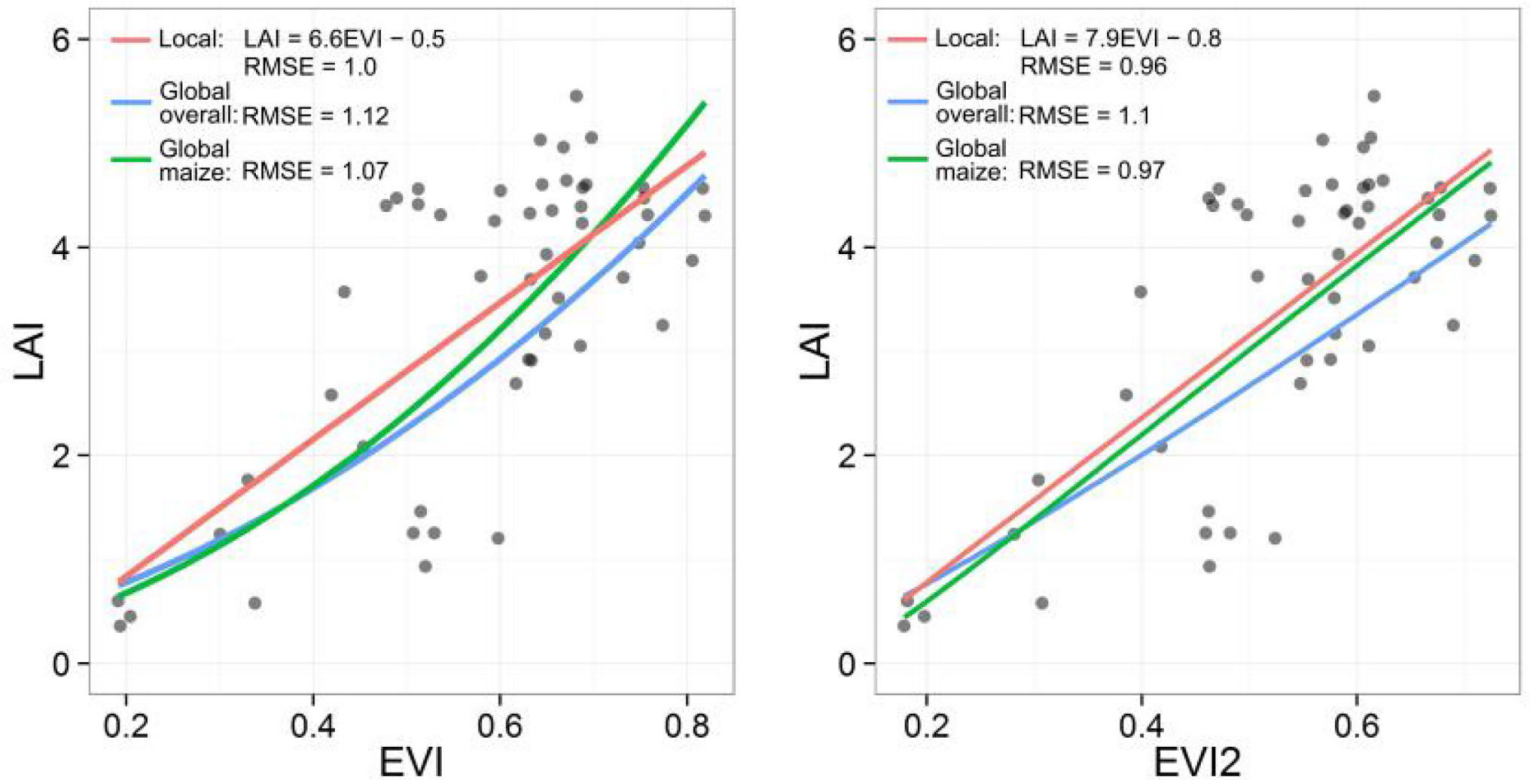
Comparison between global overall, global maize LAI-VI relationships, and local LAI-VI relationships for the maize fields in Wisconsin. The VIs were calculated based on images collected by the air-borne sensor.

At field scale (two maize fields in Wisconsin), both the global overall LAI-VI relationships and the global maize LAI-VI relationships agree well with the field measured LAI (RMSE ~1 m^2^/m^2^) and accord with local LAI-VI relationships (Figure 9). The RMSE of both global relationships are also close to that of the local relationship, meaning that the global models hold well in this validation site. The MAPE of the local relationships are 39% and 36% respectively, while those of the global relationships range from 31% to 39%. Note that the local relationships also have uncertainties from a couple of sources, including LAI measurement, linear interpolation of LAI, as well as MAC sensor calibration, and thus induces errors. In this case, the global relationships do perform well with only ~0.1 increase in RMSE.

**Figure 10:**
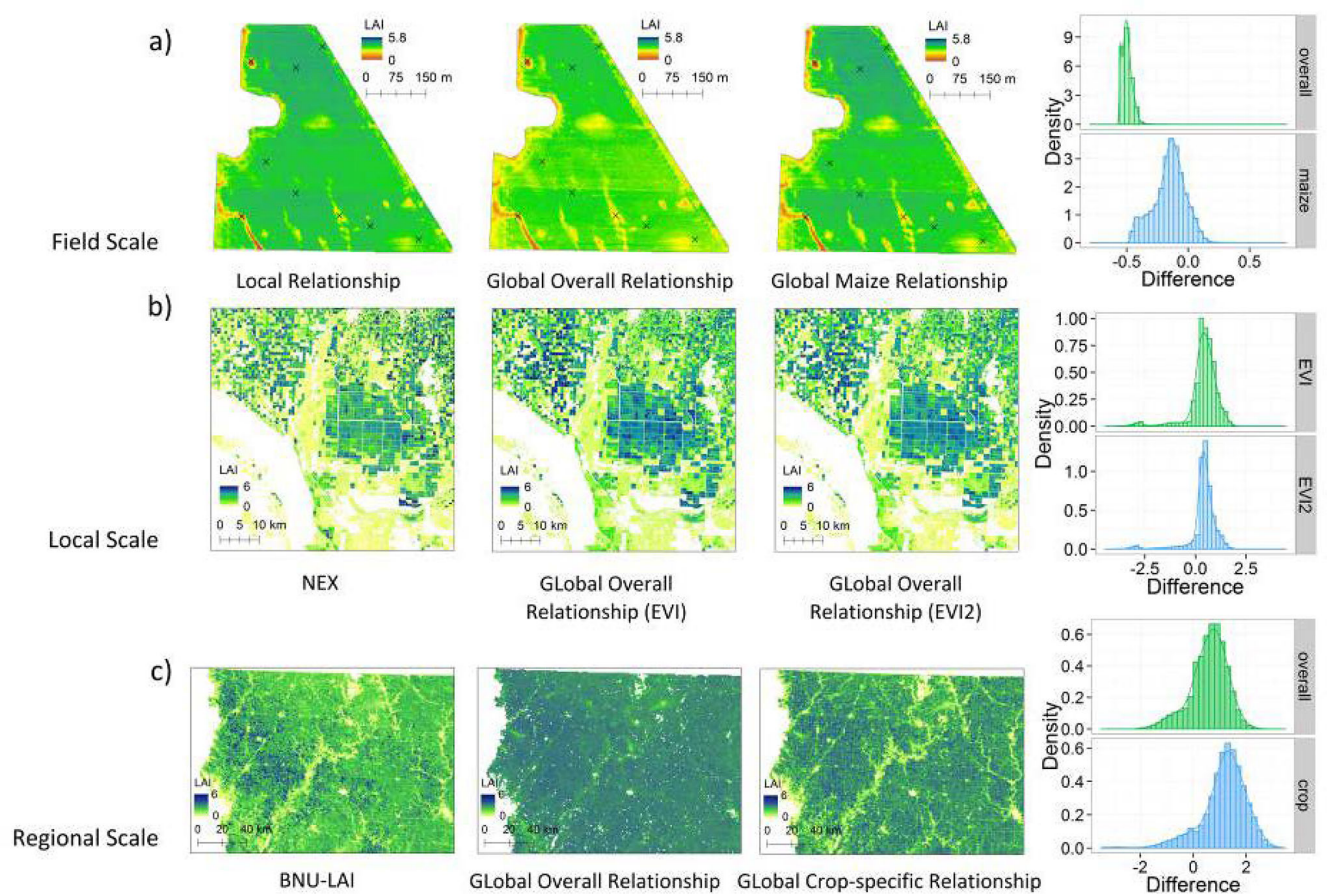
LAI maps generated using the global and local LAI-VI relationships for three example applications. In each row, the leftmost map is the reference LAI data set. The second and third maps were produced based on global LAI-VI relationships. Any LAI value greater than 6 m^2^/m^2^ is excluded from this analysis to avoid extrapolation. The fourth figure in each column shows the histograms of the difference between LAI maps based on global LAI-VI relationship and the reference map (estimated LAI minus reference LAI). Row (**a**): Results of the field scale validation in Wisconsin based on LAI-EVI relationships. The cross symbols indicate the location of LAI measurement points in the field. Row (**b**): Local scale validation in California. The maps only show a window of the footprint to highlight spatial details. The histogram of image difference was produced from a random sample of 100,000 points placed on the entire Landsat footprint. Row (**c**): Regional scale validation results in Iowa using LAI-EVI relationships. Both the reference and the map produced from this work used 16-day composite MODIS product centered on 12 July 2009.

These relationships were also used to produce LAI maps, which gave very similar spatial distribution of LAI and captured significant spatial variability in LAI associated with oxygen and water stress (Figure 10a; stress regimes described in detail in [64]). The per-pixel differences between LAI values derived from global and local relationships are within ± 0.5 m^2^/m^2^ (as shown by the histogram in Figure 10a), which is ~10% of the observed range of LAI in the study area. The MAPE between the global overall relationship and local relationship is 17%, and that of the global maize relationship is 5%. We find a slight shift in the histogram when applying the global overall relationship to this site (~0.45 m^2^/m^2^), which indicates a small bias in the global overall relationship.

#### 3.5.2. Local Scale

Local scale results were displayed in the same manner as the field scale case in Figure 10b. All maps were generated from the same Landsat image, showing only a window of the study area in California. The histograms were produced from 10,000 random samples extracted from the whole study area. Although the landscape in this area is heterogeneous with various crop fields, maps produced with the global relationships and the NEX method exhibit similar spatial distribution. A figure of 86% of the LAI estimation from global relationships is within 1 m^2^/m2 difference from NEX LAI. The MAPE of the global relationships is around 27%. The LAI generated from global relationships were generally higher than the NEX LAI, as the histogram of differences is shifted by 0.4–0.5 right to the zero point (Figure 10b).

Beside, we also used the SMEX02-WC data (not included in the model establishment) to validate the global relationships. The SMEX02-WC contains 40 maize and 20 soybean LAI data collected in Iowa, 2002. The RMSE for the LAI-EVI overall, maize, and soybean relationships are 0.69, 0.93, and 0.67 respectively, while the MAPE are 30%, 28%, and 17% respectively. The RMSE for the LAI-EVI2 overall, maize, and soybean relationships are 0.76, 1.03, and 0.64, and the MAPE are 34%, 33%, and 19% respectively.

#### 3.5.3. Regional Scale

In Figure 10c, LAI maps produced using global relationships display similar spatial distribution to that of the BNU MODIS LAI: a quite homogeneous region occupied mainly by crop fields, with very high LAI values located in the west part and low LAI values appearing around cities and along rivers, where mixed pixels mainly occur. LAI estimated using global relationships were generally higher than MODIS BNU LAI, with difference between 0 and 2.5 m^2^/m^2^. The MAPE between the LAI estimated from global relationships and MODIS LAI is around 35%. Of the two maps of global relationships, the LAI generated using global overall relationship is lower than that using global crop-specific relationship (i.e., a weighted combination of maize and soybean LAI-VI relationships).

**Figure 11:**
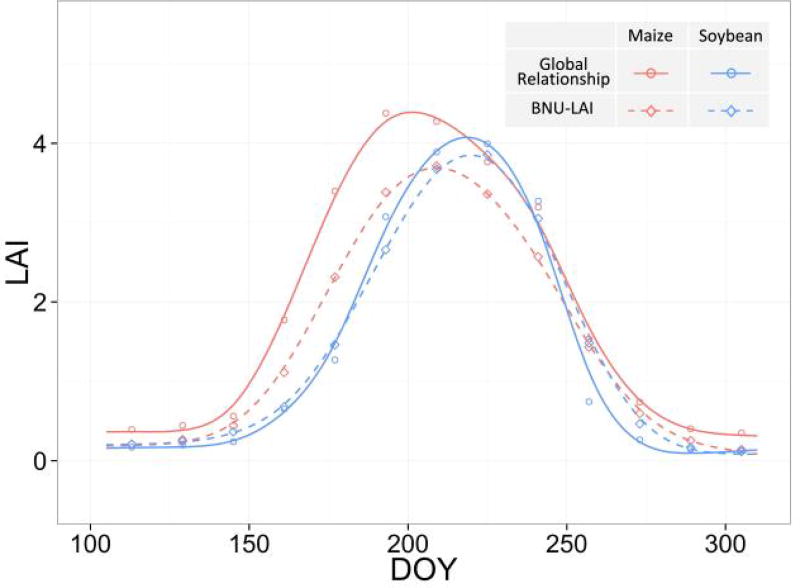
LAI time series of pure maize and soybean pixels in the Iowa validation site. LAI estimated by both the global EVI relationship and the BNU-LAI product are shown. Pure maize or soybean pixels are those that have more than 90% area planted with maize or soybean respectively. The figure shows the average value of all pure pixels. The smooth curves were generated from polynomial regression.

In this regional scale example, we performed an additional analysis by constructing crop-specific LAI time series to further compare the two datasets (Figure 11). The phenological differences between maize and soybean are well captured by both the global relationship and MODIS BNU LAI products: maize was planted 1–2 weeks earlier than soybean. In particular, the soybean LAI time series based on the global relationship agrees well with those of the MODIS BNU product. However, maize LAI predicted by the global relationship is higher than the BNU LAI. Typically, maize has a higher peak LAI than soybean at the end of the vegetative stage. This phenomenon is captured by the global LAI-VI relationship but not by the BNU LAI products, where the peak LAI of maize is even lower than that of soybean. This suggests that LAI estimates from our global LAI-VI relationships might be closer to reality than the BNU LAI products at this scale of analysis.

## 4. Discussion

### 4.1. Universality vs. Diversity

The global LAI dataset allowed us to analyze the universality and diversity of crop LAI-VI relationships quantitatively. Regardless of crop types, field locations, and time, we found that there is always a positive, monotonically increasing relationship between LAI and VIs, which explains more than half of the field measured LAI. The site-based evaluation suggests that, at a global scale, LAI-VI relationships provide an average estimate of LAI, while contributions from all other characteristics of canopy, soil, and all other environmental interactions towards the relationship are treated as random errors. Additionally, by applying the global relationships to remote sensing data of different spatial resolutions at different spatial scales, we found the global relationships to be rather robust with small discrepancies when compared to reference LAI data. To this end, the global LAI-VI relationships built in this paper are universal with an uncertainty level at RMSE of ~1 m^2^/m^2^. Despite a certain level of universality, the diversity and uniqueness of the site-specific LAI-VI relationships are real in nature. Such diversity comes from a variety of sources, including crop type [58], biochemical properties of the leaf/canopy [42,55,59], biophysical properties of the leaf/canopy [70], the optical properties of the soil background, as well as the atmospheric scattering and absorption and solar-object-sensor geometry [71]. These factors all contribute to the reflectance of crop canopy and thereafter VI. As a result, each set of environmental conditions has yielded a unique local LAI-VI relationship that is ideally applicable only under unique conditions. The diverse set of relationships between local LAI and VI introduce systematic differences in the global LAI-VI relationship with bias up to 2 m^2^/m^2^ and percentage errors between 10% and 60% when applied to local scales. In the site-based evaluation (Figure 7) and preliminary validation efforts (Figure 10), we show that such bias, or namely the diversity of local LAI-VI relationships, is inherent in the random error term of the regression model for global LAI-VI relationship.

In tandem, these results indicate that the global relationships determined here represent a powerful and useful tool for estimating LAI over large spatial scales with a predicted accuracy of ~1 m^2^/m^2^. This is particularly valuable for global applications or data-sparse regions where in situ LAI is unavailable or crop type is unknown. However, these relationships are unable to replace local relationships where available, as the local relationships take into account site-specific properties influencing the LAI-VI relationship.

### 4.2. Considerations in the Validation of the Global LAI-VI Relationships

As a preliminary validation effort, the three examples we show in Section 3.5 represent a small sample intended to demonstrate the potential strengths and limitations of our global relationships to the types of local analyses other researchers may conduct. In each of these examples, the difference histograms all demonstrate some degree of bias between the global LAI-VI relationships and reference LAI data (Figure 10). As indicated earlier, the reference data are modeled in nature, and therefore could contain errors; thus, the discrepancy between our estimates and the reference data is not necessarily “error”, and both our global LAI-VI relationships and the reference data may have contributed to the observed bias.

Ideally, to validate the global relationships, we need to draw a random sample of croplands across the globe, where we collect in-situ LAI measurements and satellite observations simultaneously. In this case, the errors are expected to possess a Gaussian distribution centered at zero, assuming that our global LAI dataset is statistically representative of the population. However, such comparison is neither practical nor possible, as in situ LAI observations are rare even at local scales. Moreover, in situ LAI were likely measured using different sampling schemes and instruments, rendering such comparison even more challenging [2]. Nevertheless, the research community has made great efforts in the validation of global satellite products with limited sources of ground truth data and various quality control and inter-comparison techniques [127,128]. As more and more ground truth data are made available, we will continue our endeavor in the validation of the global relationships.

### 4.3. Potential Issues Related to Data Quality and Consistency

There are also potential issues of the quality and consistency of the LAI and remote sensing data in this study. For example, although we expended considerable effort to gather and compile a globally representative dataset of LAI measurements and VI data, the sample size and their geographic distribution are far from ideal. Most of the maize data are from the U.S., so major producers in Asia (e.g., China) are underrepresented. The sample size of rice is small due to lack of data in major rice producing nations such as India. Inclusion of additional LAI data from underrepresented areas and crop types would undoubtedly strengthen the universality analysis of the relationships presented here.

The lack of data consistency is the most significant problem when pooling together in situ LAI data from different experiments and sites, which is common in remote sensing inter-comparison and validation work [100]. The inconsistency in measurement method is one of the potential issues. For example, optical methods using transmittance to estimate green LAI is contested in the remote sensing community [14,17,98,99]. In this paper, we show that the optical methods might yield underestimation of LAI using a residual analysis after Theil-Sen regression. Note that such underestimation might be attributed to many other factors besides the uncertainty in the optical methods. For example, in the dataset, the average value of LAI measured destructively is greater than that of LAI2000 and AccuPAR (Table 2). Moreover, since Theil-Sen regression is a robust method suppressing influences of outliers, comparison of all residuals might be biased without considering outliers. In our global dataset, some experiments/sites, such as the VALERI sites (Table S1) had applied rigorous calibration procedure for optical methods considering clumping index, gap fraction, and leaf angle etc., which renders the optically measured LAI more reliable and inter-comparable between different sites [19]. However, such calibration effort is not always available across the globe. Therefore, our study represents a valuable step towards understanding the consistency of different measurement methods at a global scale.

Beyond measurement methods, there are many other issues of inconsistency in in situ LAI data, such as the definition of LAI, sampling scheme, and design of sample plots, which all make it more challenging to compare observed LAI from different experiments and sites and relate them to remote sensing observations. Fortunately, great strides are being made by the remote sensing community to standardize in situ measurements and protocols for robust validation of LAI [2,19]. One example is the VALERI project, which consists of a number of sites representing different biomes in Europe and part of Asia. This project provides robust and concurrent methodology in site selection, establishment of elementary sampling unit (ESU), in situ measuring and calibration procedure, as well as spatial transfer function to relate locally measured biophysical variables including LAI to high resolution satellite images [19]. In addition, progress has been made towards proposing best practices in generating and validating remote sensing LAI products by the Committee on Earth Observation Satellites (CEOS) Working Group on Calibration and Validation (WGCV) Land Product Validation (LPV) sub-group [2]. This group provides a protocol of “good practices” in collection of in situ reference LAI. These efforts are intended to help standardize in situ measurement and validation processes from a remote sensing perspective and promote global synthesis and collaboration towards the understanding of remote measurement of LAI from space.

Besides the LAI in situ measurements, remote sensing products also have quality issues. One example is the mixed pixel problem, which is more common with coarse spatial resolution observations (e.g., MODIS) than Landsat images [102,103]. We noticed that many LAI measurements were made close to field edges and roads, due to accessibility. As a result, some measurements may correspond to pixels that are a mixture of crop and non-crop signals. Another source of error is the effect of Sun-sensor geometry on bidirectional surface reflectance [104–106]. For a study with a global scope and a large temporal spanning, the variations in view-illumination geometry could vary significantly from one site to another. We have considered this effect by applying MODIS NBAR product which contains BRDF-corrected data in the validation case in Iowa. We did not apply BRDF-correction to the Landsat data, since our analysis on the variation of Sun illumination geometry of the Landsat images showed that the effect of BRDF is minimal (Appendix G). Additionally, the uncertainty in the radiometric/atmospheric corrections of the satellite images is also a factor affecting data quality.

### 4.4. Concerns in the Model Prediction Power

All of the VIs are shown to have little sensitivity to LAI values above 6 m^2^/m^2^, resulting in severe saturation issues in NDVI, EVI, and EVI2, for overall and crop-specific relationships except wheat. This affects the prediction power of the LAI-VI relationships, as VIs or spectral reflectance might not be able to detect any change in LAI for very dense canopies. Interestingly, unlike the other models, the wheat crop LAI-VI relationships provide reasonable predictions over the full range (0–8.5 m^2^/m^2^), as peak LAI above 7 m^2^/m^2^ is commonly found in various wheat growing locations around the world [36,65]. In this work, we present two versions of the LAI-VI relationships derived from full-range or truncated sample, and consider both to be valid. Note that the relationships based on truncated samples have a valid range up to 6 m^2^/m^2^, and such cut-off is not uncommon in satellite-derived LAI products. For example, the CYCLOPES LAI product derived from SPOT-Vegetation has a valid LAI range of 0–6 m^2^/m^2^, as the algorithm relies on radiative transfer model simulations with certain LAI value range [127,129]. The GEOV1 global LAI product has a limit up to 7 m^2^/m^2^ [130]. Readers are advised to be aware of the differences in the two versions of relationships, and cautious should be exercised when interpreting the results, and applying the models.

Different VIs also present differed sensitivity to across different LAI value ranges (within 0–6 m^2^/m^2^). Some (e.g., NDVI, EVI, and EVI2) are more sensitive to LAI before 2 or 3 m^2^/m^2^, perhaps because they are more resistant to soil background, and others (e.g., SR, CI_Green_) are more sensitive after 3 m^2^/m^2^, since they have less saturation effect (e.g., Figure 3). Previous studies have found improved predicting power by choosing VIs on different LAI value ranges according to sensitivity [67,131]. This is a potential solution to reduce the uncertainty of the global relationships and deserve further investigations.

## 5. Concluding Remarks

In this study, we developed a dataset containing spatiotemporally explicit in situ crop LAI measurements gathered worldwide to assess the global universality of LAI-VI relationships. In the exploratory analysis, we built best-fit functions between LAI observations and five vegetation indices (SR, NDVI, EVI, EVI2, and CI_Green_) generated from Landsat data to depict global LAI-VI relationships for a number of crop types. Results reveal that the global LAI-VI relationships explain more than half of the variance in field-measured LAI using only remotely sensed observations. The LAI-VI relationships are crop specific and are most effective using EVI or EVI2 from surface reflectance. To account for measurement errors from both LAI and VIs, we further refined the EVI and EVI2 models using power transformations and Theil-Sen estimator and the final models have RMSE mostly below 1.0 m^2^/m^2^. We provided three examples that applied the global LAI-EVI/EVI2 relationships to local to regional spatial scales, and found them to be effective in generating LAI maps. Based on the preliminary validation and site-based evaluation, we found that the LAI-VI relationships we built possess global university, with random errors reflecting the diverse nature of agro-ecosystem landscapes.

The major contributions of this work include synthesizing a large number of in situ LAI observations and vegetation indices from various locations and developing a set of globally applicable statistical relationships. The simplicity of generating VI using remotely sensed images and applying simple statistical relationships adds to the practical value of this research, especially when essential variables needed for process-based methods are rarely present and hard to measure [27,107]. Moreover, to the best of our knowledge, the work presented here is the first to compile a large global dataset of crop LAI and VIs and analyze the universality and diversity of the LAI-VI relationships globally. These findings not only support the CEOS Land Product Validation framework for the validation of remote sensing LAI products but also contribute to a larger community of users that are interested in producing LAI maps from remote sensing but do not have access to measured LAI data [58,93,101]. Moreover, as our analysis was based on Landsat images with 30 meter spatial resolution, the global LAI-VI relationships support production of a large scale fine resolution LAI map which is essential to agricultural applications, especially in regions where crop fields are relatively small. The ability to produce LAI maps at this level provides potentials to assess additional biophysical variables and processes including biomass, primary production (NPP), evapotranspiration, and crop yields, at individual plot/field level, which is more suitable for decision making than aggregated values over a large area. The easy accessibility, low cost, and the long historical coverage and continuity of Landsat mission also render our findings useful to scientific, governmental, and commercial applications. Finally, the analysis and findings here only apply to broadband VIs. As more and more medium to high resolution sensors become available with additional narrow spectral bands, i.e., the red edge band, there will be great opportunities of establishing efficient models for global LAI estimation with various hyperspectral VIs [58,132,133].

## Supplementary Material

Supp1

## Figures and Tables

**Table 1:** Vegetation indices used in this research (NIR, Red, Green, and Blue corresponds to TM/ETM+ band 4, band 3, band2, and band 1 respectively).

Index	Equation
Simple Ratio	$\text{SR}=\frac{\text{NIR}}{\text{Red}}$
Normalized Difference Vegetation Index	$\text{NDVI}=\frac{\text{NIR}-\text{Red}}{\text{NIR}+\text{Red}}$
Enhanced Vegetation Index	$\text{EVI}=2.5\frac{\text{NIR}-\text{Red}}{1+\text{NIR}+6\text{Red}-7.5\text{Blue}}$
Enhanced Vegetation Index 2	$\text{EVI}2=2.5\frac{\text{NIR}-\text{Red}}{1+\text{NIR}+2.4\text{Red}}$
Green Chlorophyll Index	${\text{CI}}_{\text{Green}}=\frac{\text{NIR}}{\text{Green}}-1$

**Table 2:** Statistics of leaf area index (LAI) dataset by crop type, measurement method, and region.

	Count	LAI (m^2^/m^2^)			

		Mean	Std.	Min	Max
Overall	1459	2.52	1.62	0.10	6.00
*By crop types*					
Maize	366	3.08	1.51	0.12	5.98
Soybean	90	2.13	1.37	0.10	5.51
Wheat	261	2.78	1.62	0.10	6.00
Rice	44	3.35	1.86	0.12	5.98
Cotton	95	2.20	1.74	0.11	5.79
Pasture	263	1.97	1.51	0.10	5.95
*By measurement methods*					
Destructive	235	2.88	1.73	0.10	5.98
LAI2000	692	2.16	1.47	0.10	5.98
AccuPAR	375	2.71	1.70	0.11	5.98
Hemispheric	157	3.15	1.52	0.10	6.00
*By geographical region*					
US	501	2.60	1.73	0.10	5.98
Europe	668	2.90	1.56	0.10	6.00
Asia	14	2.90	1.59	0.30	5.98
Australia	272	1.41	0.98	0.10	4.84

**Table 3:** LAI-EVI and LAI-EVI2 relationships (*LAI* = *f*(*VI*)) based on data transformation and simple linear regression (SLR) with Theil-Sen estimator. The confidence interval of the slope estimates was calculated using analytical solutions by Sen [92].

Crop Type	VI	SLR model	Coefficient (Confidence Interval)		Prediction Model	RMSE (m^2^/m^2^)	MAE (m^2^/m^2^)	Quantiles of Absolute Residuals (m^2^/m^2^)				
	
			a	b				5%	25%	50%	75%	95%
**Overall**	EVI	$\sqrt{y}=\text{ax}+b$	2.07 (1.97, 2.17)	0.47	***y*** = (***ax*** + ***b***)^**2**^	1.13	0.89	0.06	0.33	0.70	1.38	2.24
	EVI2	$\sqrt{y}=a\sqrt{x}+b$	2.92 (2.78, 3.06)	−0.43	$y={\left(a\sqrt{x}+b\right)}^{2}$	1.11	0.87	0.06	0.32	0.70	1.33	2.17

**Row crop**	EVI	$\sqrt{y}=\text{ax}+b$	2.16 (2.1, 2.32)	0.41	***y*** = (***ax*** + ***b***)^**2**^	1.14	0.89	0.06	0.31	0.67	1.32	2.29
	EVI2	$\sqrt{y}=a\sqrt{x}+b$	3.16 (3.01, 3.31)	−0.58	$y={\left(a\sqrt{x}+b\right)}^{2}$	1.12	0.86	0.06	0.30	0.67	1.28	2.22

**Maize**	EVI	$\sqrt{y}=\text{ax}+b$	2.42 (2.21, 2.65)	0.34	***y*** = (***ax*** + ***b***)^**2**^	1.01	0.81	0.07	0.33	0.72	1.15	1.98
	EVI2	$y^{\frac{2}{3}}=a\sqrt{x}+b$	5.3 (4.89, 5.68)	−1.66	$y={\left(a\sqrt{x}+b\right)}^{\frac{3}{2}}$	0.92	0.74	0.06	0.29	0.65	1.02	1.81

**Soybean**	EVI	$\sqrt{y}=\text{ax}+b$	2.53 (2.28, 2.76)	0.08	***y*** = (***ax*** + ***b***)^**2**^	0.69	0.49	0.02	0.14	0.32	0.68	1.45
	EVI2	$\sqrt{y}=\text{ax}+b$	2.77 (2.47, 3.03)	0.06	***y*** = (***ax*** + ***b***)^**2**^	0.70	0.51	0.04	0.15	0.34	0.78	1.42

**Wheat**	EVI	$y^{\frac{3}{4}}=\text{ax}+b$	4.24 (3.71,4.78)	0.22	$y={\left(\text{ax}+b\right)}^{\frac{4}{3}}$	1.13	0.94	0.07	0.41	0.82	1.34	2.03
	EVI2	$y^{\frac{3}{4}}={\text{ax}}^{\frac{3}{5}}+b$	5.47 (4.81, 6.16)	−1.03	$y={\left({\text{ax}}^{\frac{3}{5}}+b\right)}^{\frac{4}{3}}$	1.13	0.94	0.12	0.41	0.87	1.37	2.12

**Rice**	EVI	$y^{\frac{2}{3}}=\text{ax}+b$	4.27 (3.25, 5.23)	−0.05	$y={\left(\text{ax}+b\right)}^{\frac{3}{2}}$	1.03	0.79	0.07	0.35	0.67	1.02	2.38
	EVI2	$y^{\frac{3}{4}}=\text{ax}+b$	5.32 (4.08, 6.51)	−0.18	$y={\left(\text{ax}+b\right)}^{\frac{4}{3}}$	1.02	0.78	0.06	0.34	0.70	1.06	2.35

**Cotton**	EVI	$\sqrt[{3}]{y}=a\frac{1}{\sqrt[{3}]{x}}+b$	−1.25 (−1.39, −1.11)	2.97	$y={\left(a\frac{1}{\sqrt[{3}]{x}}+b\right)}^{3}$	0.91	0.73	0.05	0.25	0.55	1.12	1.62
	EVI2	$\sqrt[{3}]{y}=a\frac{1}{\sqrt[{3}]{x}}+b$	−1.21 (−1.33, −1.07)	2.95	$y={\left(a\frac{1}{\sqrt[{3}]{x}}+b\right)}^{3}$	0.93	0.76	0.04	0.33	0.64	1.16	1.61

**Pasture**	EVI	$y^{\frac{3}{4}}={\text{ax}}^{2}+b$	2.84 (2.49, 3.20)	0.88	$y={\left({\text{ax}}^{2}+b\right)}^{\frac{4}{3}}$	0.98	0.81	0.10	0.45	0.72	1.07	2.00
	EVI2	$y^{\frac{3}{4}}={\text{ax}}^{\frac{3}{2}}+b$	2.99 (2.6, 3.37)	0.72	$y={\left({\text{ax}}^{\frac{3}{2}}+b\right)}^{\frac{4}{3}}$	0.99	0.82	0.06	0.42	0.70	1.12	1.91
