# Supplementary material for: How Universal Is the Relationship between Remotely Sensed Vegetation Indices and Crop Leaf Area Index? A Global Assessment
Source: Remote Sens (Basel). Author manuscript; Available in PMC 2018 Jul 10. (PMC6038712; doi:10.3390/rs8070597)
Supplement: Supp1 [file NIHMS948329-supplement-Supp1.pdf]

Supplementary Materials: How Universal Is the Relationship between Remotely Sensed Vegetation Indices and Crop Leaf Area Index? A Global Assessment

Yanghui Kang, Mutlu Özdoğan, Samuel C. Zipper, Miguel O. Román, Jeff Walker, Suk Young Hong, Michael Marshall, Vincenzo Magliulo, José Moreno, Luis Alonso, Akira Miyata, Bruce Kimball and Steven P. Loheide II

1. List of LAI Measurement Sites

Table S1. Summary of LAI measurement sites and records.

Site Name	Source	Country	Major Crops	No. of Records	No. of Records in Final Analysis
Agro	Bigfoot [1]	US	Soybean, Maize	290	0
Beltsville	Collaboration	US	Maize	26	26
Mead	AmeriFlux [2]	US	Maize	145	127
California	Collaboration	US	Alfalfa, Cotton, Maize, Rice	270	223
Maricopa	DSSAT [3–5]J	US	Cotton	18	18
Missouri	Collaboration [6]	US	Maize, Soybean	50	37
SMEX02-IA	SMEX02 [7]	US	Maize, Soybean	39	39
SMEX02-WC	SMEX02 [7]	US	Maize, Soybean	60	0
SMEX03-GA	SMEX03 [8]	US	Cotton	15	7
SMEX03-OK	SMEX03 [9]	US	Maize, Alfalfa, Soybean	25	24
Brazil	DSSAT [3–5]	Brazil	Maize	6	4
Les Alpilles	VALERI [10]	France	Tomato, Alfalfa	47	41
Sud-ouest	VALERI [10]	France	Maize, Soybean, Sunflower	24	19
Gilching	VALERI [10]	Germany	Wheat, Maize	13	5
Fundulea	VALERI [10]	Romania	Wheat, Alfalfa	21	13
Barrax	VALERI [10]	Spain	Alfalfa, Maize, Sugar beet	34	32
AGRISAR	ESA EO Campaigns Data (WWW2) [11]	Germany	Wheat, Sugar beet	211	181
CEFLES2	ESA EO Campaigns Data [11]	France	Maize, Wheat	58	47
SEN2FLEX	ESA EO Campaigns Data [11]	Spain	Maize	6	6
SEN3EXP2009	ESA EO Campaigns Data [11]	Spain	Maize, Sunflower, Garlic	37	26
SPARC	ESA EO Campaigns Data [11]	Spain	Pasture, Maize, Sugar beet	288	263
Italy	CarboEurope [12]	Italy	Maize	41	35
Mase	AsiaFlux [13]	Japan	Rice	4	3
IRR	AsiaFlux [13]	Philippines	Rice	9	5
China	Publication [14]	China	Winter wheat	8	6
AACES1	AACES1 [15]	Australia	Wheat, Fallow	7	5
AACES2	AACES2 [15]	Australia	Wheat, Oat, Pasture	10	4
NAFE05	NAFE05 [16]	Australia	Wheat, Barley	8	7
NAFE06	NAFE06 [17]	Australia	Wheat, Pasture, Barley	107	101
SMAPEx2	SMAPEx2 [18]	Australia	Maize, Wheat, Pasture	48	0
SMAPEx3	SMAPEx3 [18]	Australia	Wheat, Barley, Pasture	161	155
			Total	2086	1459

2. Removal of Three Sites

2.1. Agro Site

The Agro site is a valuable data source which was designed for remote sensing product validation, and has been utilized in many satellite imagery oriented investigations [19,20]. However, we did not detect any statistically significant relationships between LAI and VI extracted from Landsat images when pooling data collected at different times, which violates a fundamental assumption of our study (Figure S1). Moreover, we found nearly 1/3 of the maize LAI measures are greater than 8 m^2/m^2, which is beyond the prediction power of satellite derived VIs. Due to the lack of necessary metadata to resolve these issues and the large sample size at Agro (*n* = 290), we elected to eliminate the Agro site from the truncated dataset, but retain it in the full-range dataset.

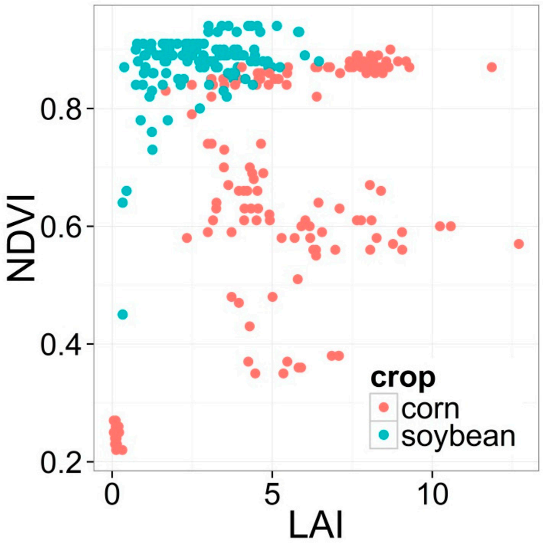

Figure S1. Scatterplot of surface reflectance derived NDVI versus LAI measurements of the Agro Site (colored by crop types).

2.2. SMEX02-WC Site

The SMEX02-WC site was excluded due to presence of unusually high value of the CI_{Green} calculated from surface reflectance. SMEX02-WC is located in Iowa, US. This site contains two types of crops: maize and soybean. Figure S2 shows the distribution of CI_{Green} (surface reflectance based) values for SMEX02-WC vs. the rest of the dataset. Over 50% of the SMEX02-WC CI_{Green} measurements were above 15, while the rest of the dataset as well as literature reported values for CI_{Green} range from 0.5 to 15 [21–25]. Thus, due to remotely sensed data quality concerns, we eliminated all data from this site in the establishment of the dataset, but used it to validate the refined LAI-EVI/EVI2 relationships.

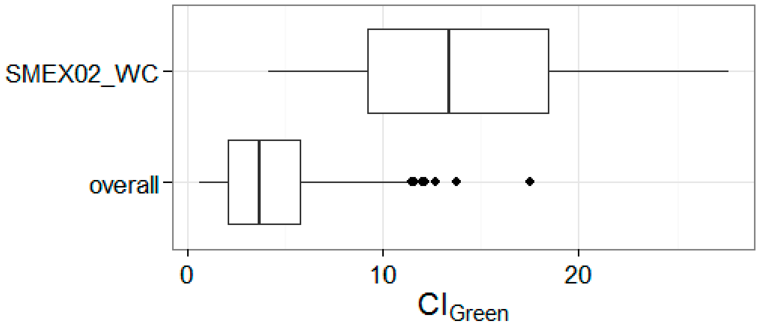

Figure S2. Boxplot of the CI_{Green} values of SMEX02-WC versus rest of the dataset.

2.3. SMAPEx2 Site

SMAPEx2 (Australia) contains 19 samples in pasture and 29 samples in cereals such as wheat and barley. It was eliminated as many of the measurements were taken during crop reproductive stage. Figure S3 shows a scatter plot of LAI versus EVI, colored by crop type. Although an overall relationship considering all crops is significant (R^2 = 0.18), there is no significant relationship for pasture and row crop data if treated separately ($R^2 \le 0.02$). In the figure, pasture LAI varies from 0–2 m^2/m^2. However, EVI stays around 0.18, and never rises above 0.21. The same behavior is also found in more than half of the row crop samples.

To explain the behavior of LAI in SMAPEx2, we investigated the photos taken for each record. Figure S4 shows two pasture fields at the time of measurement in SMAPEx2. Although plot YD_F606 has a much greater LAI (1.6 m^2/m^2) than plot YA4_F14 (0.56 m^2/m^2), the average EVI for both plots are almost the same. This is because the pasture in YD_F606 is mainly mature oats, with most leaves being yellow, which seem to be included in the measured LAI using an optical instrument such as LAI2000. The same issue also presents with cereals. Therefore, the definition of LAI used in SMAPEx2 might be different from the definition we used which contains only "green" leaves. We also found that the SMAPEx2 experiment was conducted in early December 2010. Hence most of the grains were at maturity and turned yellow (Figure S5). The case for pasture is more complicated than grains, as it may contain a variety of plants, which mature at different time of the year. Without aids from photos taken in the field, it is difficult to determine or prove whether each LAI measures include yellow leaves or not. Therefore, we removed all data from SMAPEx2 as a precaution.

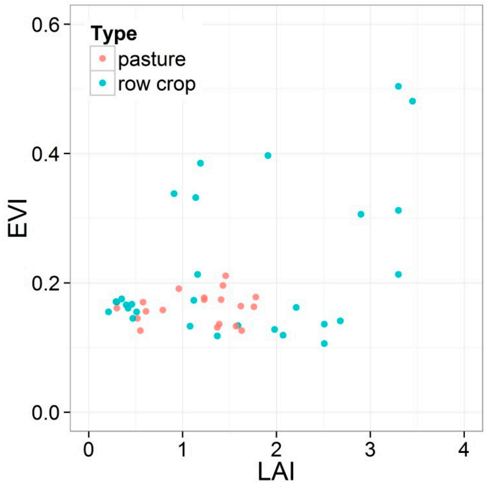

Figure S3. Scatterplot of surface reflectance derived EVI versus LAI for SMAPEx2 (colored by crop type).

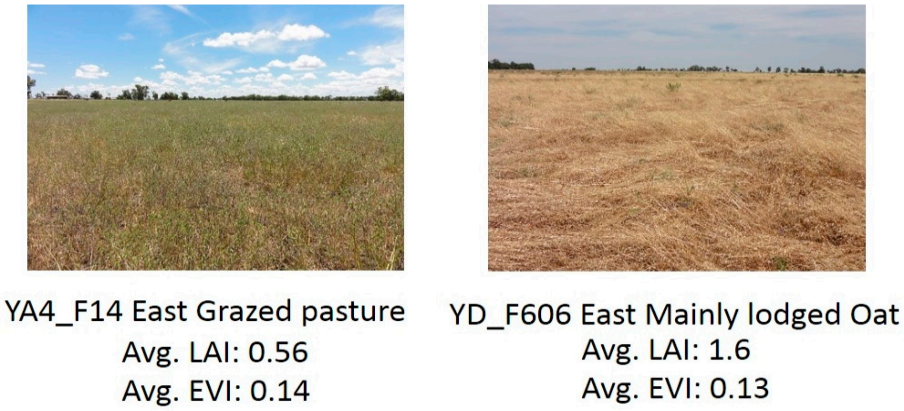

Figure S4. Field photos of two pasture plots in SMAPEx2. The average LAI and EVI values are also provided for data within each plot.

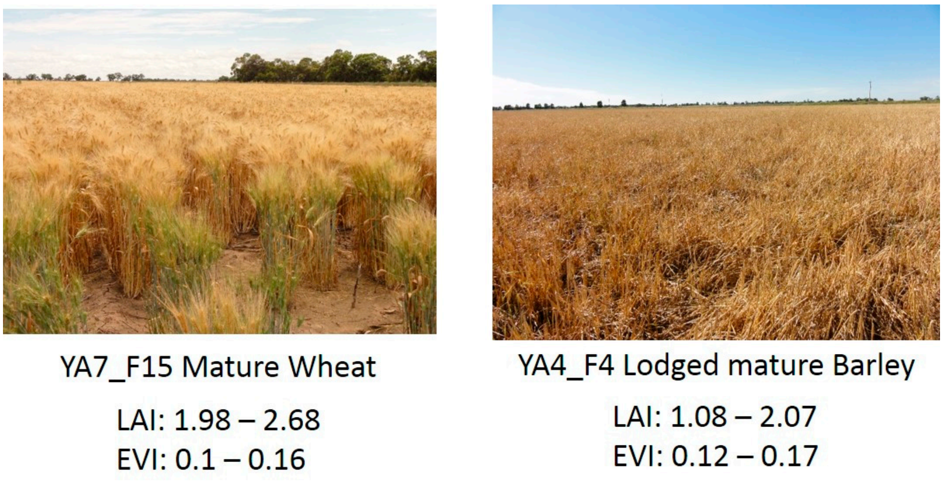

Figure S5. Field photos of two grain plots in SMAPEx2. The average LAI and EVI values are also provided for data within each plot.

3. List of Tested VIs in Selected Literatures

Table S2. VIs used in literatures that established LAI-VI relationships for crop (only broadband VIs are shown).

Literature	Sensor/Simulation	Crop Type	Tested VIs	Best VIs
Baret, F. & Guyot, 1991 [19]	SAIL model	General crop canopy	NDVI, PVI [a], SAVI, TSAVI [b]	-
Casanova et al., 1998 [61]	Ground spectrometer	Rice	SR, NDVI, PVI, WDVI	PVI, WDVI
Broge & Leblanc, 2001 [42]	PROSPECT + SAIL model	General crop canopy	SR, NDVI, PVI, WDVI [c], SAVI, TSAVI, ATSAVI [d], SAVI2, MSAVI2	NDVI and SR at low LAI values, MSAVI2 at high LAI values
Colombo et al., 2003 [62]	IKONOS	Maize, soybean	NDVI, SR, SAVI, PVI, ARVI [e], EVI	NDVI
Gitelson, 2004 [55]	Ground spectrometer	Maize, soybean, wheat	NDVI, WDRVI [f]	WDRVI
Viña et al., 2011 [57]	Ground spectrometer	Maize, soybean	SR, NDVI, EVI, GARI [g], WDRVI, CIGreen	CIGreen
Guindin-Garcia et al., 2012 [16]	MODIS	Maize	WDRVI, EVI	WDRVI
Liu et al., 2012 [58]	Landsat TM, ETM+	Maize, soybean, wheat	NDVI, EVI2, OSAVI [h], MTVI2 [i]	EVI2, OSAVI, and MTVI2 are better than NDVI.
Kim et al., 2012 [122]	Landsat TM, IKONOS, MODIS	Maize, soybean	NDVI	-
Nguy-Robertson et al., 2012 [66]	Ground spectrometer	Maize, soybean	SR, NDVI, Green NDVI, OSAVI, CIGreen, WDRVI, EVI2	CIGreen
Nguy-Robertson et al., 2014 [59]	Ground spectrometer	Maize, soybean, wheat, potato	SR, Green NDVI, CIGreen, WDRVI, Green WDRVI	CIGreen, Green WDRVI

[a] PVI: Perpendicular Vegetation Index; [b] TSAVI: Transformed Soil Adjusted Vegetation Index; [c] WDVI: Weighted Difference Vegetation Index; [d] ATSAVI: adjusted TSAVI; [e] ARVI: Atmospherically Resistant Vegetation Index; [f] WDRVI: Wide Dynamic Range Vegetation Index; [g] GARI: Green Atmospherically Resistant Vegetation Index; [h] OSAVI: Optimized Soil Adjusted Vegetation Index; [i] MTVI: modified triangular vegetation index.

4. Statistical Description of the Full-Range Dataset

Table S3. Statistics of the full-range LAI dataset by crop type, measurement method, and region.

	Count	LAI (m^2/m^2)			
		Mean	Std.	Min	Max
Overall	1784	2.74	1.92	0.002	8.45
By crop types					
Maize	511	3.31	2.07	0.01	8.45
Soybean	218	2.39	1.41	0.01	6.46
Wheat	298	3.18	2.04	0.002	8.22
Rice	54	3.34	2.17	0.04	7.86
Cotton	102	2.24	1.89	0.04	6.87
Pasture	267	2.02	1.51	0.030	6.80
By measurement methods					
Destructive	498	3.02	2.11	0.01	8.45
LAI2000	702	2.23	1.59	0.002	7.51
AccuPAR	419	3.05	2.05	0.04	8.22
Hemispheric	165	3.33	1.69	0.10	7.80
By geographical region					
US	780	2.85	2.06	0.01	8.45
Europe	716	3.12	1.79	0.002	8.22
Asia	18	3.75	2.16	0.30	7.86
Australia	264	1.37	0.92	0.02	4.41

5. LAI-VI Relationships based on truncated and full-range datasets

Table S4. Best-fit functions for the LAI-VI relationships ($LAI = f(VI)$) for major crops based on three levels of radiometric/atmospheric corrections. The last column gives the reasonable VI range that will produce LAI within [0,6] m^2/m^2 and any value out of that range will result in either negative of excessive (greater than 6 m^2/m^2) LAI.

		Index	Equation	Coefficient			95% Confidence Interval			Reasonable VI Range
				a	b	c	a	b	c	
**Overall**	Radiance	SR	$y = a \times \log(b \times x + c)$	2.77	0.76	0.44	(2.14,3.4)	(0.42,1.09)	(0.09,0.79)	[1,10.97]
		NDVI	$y = a \times b^x$	0.77	11.95	-	(0.7,0.84)	(10.23,13.67)	-	[0,0.83]
		CI_{Green}	$y = a \times x^b + c$	2.45	0.61	0.01	(2.09,2.8)	(0.52,0.7)	(−0.3,0.33)	[0,4.34]
	TOA Reflectance	SR	$y = a \times \log(x) + b$	2.25	−0.65	-	(2.15,2.35)	(−0.8,−0.51)	-	[1.34,19.28]
		NDVI	$y = a^x + b$	8.46	−1.18	-	(8.11,8.81)	(−1.29,−1.07)	-	[0.08,0.92]
		EVI	$y = a \times x + b$	4.71	−0.22	-	(4.49,4.93)	(−0.37,−0.08)	-	[0.05,1]
		EVI2	$y = a \times x + b$	6.91	−0.22	-	(6.56,7.26)	(−0.37,−0.07)	-	[0.03,0.9]
		CI_{Green}	$y = a \times x + b$	0.94	0.19	-	(0.9,0.99)	(0.06,0.31)	-	[0,6.17]
	Surface Reflectance	SR	$y = a \times \log(x + b) + c$	1.94	0.34	−0.91	(1.65,2.23)	(−0.36,1.04)	(−1.7,−0.12)	[1.21,32.21]
		NDVI	$y = a \times b^x$	0.26	26.28	-	(0.22,0.3)	(21.21,31.34)	-	[0,0.99]
		EVI	$y = a \times x + b$	5.67	−0.26	-	(5.39,5.95)	(−0.41,−0.11)	-	[0.04,1]
		EVI2	$y = a \times x^b$	6.21	1.16	-	(5.92,6.49)	(1.09,1.24)	-	[0,0.96]
		CI_{Green}	$y = a \times x^b + c$	2.86	0.42	−2.60	(1.45,4.28)	(0.28,0.55)	(−4.14,−1.06)	[0.76,17.17]
**Rowcrop**	Radiance	SR	$y = a \times \log(x) + b$	2.44	0.22	-	(2.32,2.56)	(0.08,0.35)	-	[1,10.72]
		NDVI	$y = a \times x + b$	5.91	0.09	-	(5.6,6.21)	(−0.06,0.24)	-	[0,1]
		CI_{Green}	$y = a \times \log(x + b) + c$	3.10	0.83	0.73	(2.37,3.82)	(0.44,1.23)	(−0.43,1.89)	[0,4.64]
	TOA Reflectance	SR	$y = a \times \log(x) + b$	2.38	−0.77	-	(2.26,2.49)	(−0.93,−0.6)	-	[1.38,17.19]
		NDVI	$y = a \times x^b + c$	7.29	2.33	0.22	(6.8,7.78)	(1.99,2.67)	(−0.03,0.47)	[0.05,0.91]
		EVI	$y = a \times x + b$	5.05	−0.37	-	(4.79,5.31)	(−0.54,−0.2)	-	[0.07,1]
		EVI2	$y = a \times x + b$	7.49	−0.38	-	(7.08,7.89)	(−0.56,−0.21)	-	[0.05,0.85]
		CI_{Green}	$y = a \times x + b$	1.02	0.08	-	(0.97,1.07)	(−0.07,0.22)	-	[0,5.81]
	Surface Reflectance	SR	$y = a \times \log(x) + b$	1.88	−0.62	-	(1.79,1.97)	(−0.78,−0.45)	-	[1.39,33.58]
		NDVI	$y = a \times x^b$	5.88	2.09	-	(5.62,6.13)	(1.94,2.24)	-	[0,1]
		EVI	$y = a \times x + b$	6.08	−0.42	-	(5.76,6.4)	(−0.59,−0.24)	-	[0.07,1]
		EVI2	$y = a \times x + b$	6.82	−0.54	-	(6.47,7.17)	(−0.71,−0.36)	-	[0.08,0.96]
		CI_{Green}	$y = a \times \log(x) + b$	2.02	−0.08	-	(1.92,2.12)	(−0.22,0.06)	-	[1.04,20.44]

Table S4. *Cont.*

		Index	Equation	Coefficient			95% Confidence Interval			Reasonable VI Range
				a	b	c	a	b	c	
**Maize**	Radiance	SR	$y=a-b^{(x+c)}$	4.74	0.60	−3.99	(4.33,5.14)	(0.52,0.68)	(−4.82,−3.17)	[1,3.53]
		NDVI	$y=a\times x+b$	5.58	0.35	-	(5,6.15)	(0.04,0.66)	-	[0,1]
		CIGreen	$y=a\times x^b+c$	9.48	0.11	−6.45	(−3.8,22.76)	(−0.05,0.27)	(−19.66,6.76)	[0.03,11.86]
	TOA Reflectance	SR	$y=a\times x^b+c$	247.93	0.01	−248.48	(−8253,8750	(−0.3,0.32)	(−8751,8254)	[1.27,16.64]
		NDVI	$y=(x+a)^b$	0.61	4.52	-	(0.57,0.65)	(4.11,4.93)	-	[0,0.88]
		EVI	$y=a\times x^b$	5.76	1.57	-	(5.46,6.05)	(1.41,1.73)	-	[0,1]
		EVI2	$y=a\times x^b$	8.67	1.35	-	(7.53,9.8)	(1.16,1.54)	-	[0,0.76]
		CIGreen	$y=a\times x^b+c$	2.05	0.67	−1.30	(0.58,3.53)	(0.38,0.97)	(−2.93,0.32)	[0.51,6.57]
	Surface Reflectance	SR	$y=a\times x^b+c$	426.98	0.004	−427.40	(−24282,25136)	(−0.24,0.25)	(−25137,24282)	[1.26,34.27]
		NDVI	$y=a\times b^x$	0.19	39.70	-	(0.13,0.26)	(23.08,56.31)	-	[0,0.93]
		EVI	$y=a^x+b$	10.91	−0.98	-	(9.99,11.83)	(−1.21,−0.76)	-	[0,0.81]
		EVI2	$y=a\times x^b$	8.58	1.56	-	(7.81,9.35)	(1.4,1.72)	-	[0,0.79]
		CIGreen	$y=a\times x^b+c$	2.06	0.54	−1.91	(0.44,3.68)	(0.3,0.77)	(-3.85,0.02)	[0.87,12.23]
**Soybean**	Radiance	SR	$y=(x+a)^b$	-0.43	0.84	-	(0.1,0.75)	(0.76,0.93)	-	[1,8.81]
		NDVI	$y=a\times x+b$	5.01	0.13	-	(4.12,5.89)	(−0.27,0.54)	-	[0,1]
		CIGreen	$y=a\times x^b+c$	2.37	0.62	−0.07	(1.5,3.24)	(0.36,0.88)	(−0.83,0.69)	[0,4.55]
	TOA Reflectance	SR	$y=a-b\times x^c$	12.79	14.05	−0.20	(−39.35,11.25)	(−0.71,0.3)	(−13.45,39.03)	[1.58,34.77]
		NDVI	$y=a^x+b$	8.19	−1.43	-	(7.11,9.27)	(−1.76,−1.1)	(7.11,9.27)	[0.17,0.95]
		EVI	$y=a^x+b$	6.26	−1.16	-	(6.13,6.39)	(−1.22,−1.11)	(6.13,6.39)	[0.08,1]
		EVI2	$y=a\times x+b$	9.19	1.64	-	(7.33,11.04)	(1.36,1.92)	(7.33,11.04)	[0,0.77]
		CIGreen	$y=a\times\log(x)+b$	2.02	0.56	-	(1.73,2.3)	(0.29,0.83)	(1.73,2.3)	[0.76,14.89]
	Surface Reflectance	SR	$y=a\times x^b+c$	563.85	0.003	−564.59	(−65785,66913)	(−0.34,0.35)	(−66914,65785)	[1.56,56.11]
		NDVI	$y=a^x+b$	6.51	−1.52	-	(5.72,7.3)	(−1.87,−1.17)	-	[0.22,1]
		EVI	$y=a\times x^b+c$	6.32	1.49	−0.31	(5.55,7.1)	(0.84,2.14)	(−1.16,0.53)	[0.13,1]
		EVI2	$y=a^x+b$	10.99	−1.25	-	(9.63,12.35)	(−1.52,−0.97)	-	[0.09,0.83]
		CIGreen	$y=a\times x^b+c$	7.90	0.18	−8.03	(−13.68,29.48)	(−0.21,0.56)	(−30.04,13.98)	[1.09,25.86]

Table S4. *Cont.*

		Index	Equation	Coefficient			95% Confidence Interval			Reasonable VI Range
				a	b	c	a	b	c	
**Wheat**	Radiance	SR	$y=a-b^{(x+c)}$	4.70	0.46	−2.88	(4.29,5.12)	(0.36,0.57)	(−3.47,−2.29)	[1,2.54]
		NDVI	$y=a\times x^{b}+c$	6.16	0.98	0.55	(5.41,6.92)	(0.64,1.33)	(-0.21,1.31)	[0,0.88]
		CI_{Green}	$y=a\times x^{b}+c$	4.10	0.34	−0.95	(1.99,6.21)	(0.16,0.53)	(−2.97,1.07)	[0.01,4.66]
	TOA Reflectance	SR	$y=a-b\times x^{c}$	5.72	7.52	−0.84	(4.01,7.43)	(6.41,8.62)	(−1.34,−0.34)	[1.38,1]
		NDVI	$y=(x+a)^{b}$	0.77	3.57	-	(0.71,0.82)	(3.16,3.98)	-	[0,0.89]
		EVI	$y=a\times x^{b}+c$	7.29	0.52	−2.36	(3.28,11.31)	(0.05,0.98)	(−6.66,1.93)	[0.11,1]
		EVI2	$y=a-b\times c^{x}$	8.05	9.55	0.16	(−13.5,−5.61)	(−0.12,0.44)	(3.07,13.03)	[0.09,0.85]
		CI_{Green}	$y=a\times x^{b}$	1.41	0.84	-	(1.21,1.61)	(0.7,0.97)	-	[0,5.65]
	Surface Reflectance	SR	$y=a-b^{(x+c)}$	4.42	0.68	−5.04	(4.06,4.78)	(0.6,0.75)	(−6.07,−4)	[1.25,3.87]
		NDVI	$y=a\times x+b$	6.02	−0.93	-	(5.2,6.83)	(−1.45,−0.4)	-	[0.15,1]
		EVI	$y=a\times x+b$	7.03	−0.33	-	(6.2,7.86)	(−0.72,0.06)	-	[0.05,0.9]
		EVI2	$y=a\times x+b$	7.86	−0.46	-	(6.91,8.8)	(−0.88,−0.05)	-	[0.06,0.82]
		CI_{Green}	$y=a\times \log(x)+b$	2.14	0.12	-	(1.88,2.4)	(−0.23,0.47)	-	[0.95,15.66]
**Rice**	Radiance	SR	$y=a\times x+b$	0.67	0.18	-	(0.51,0.84)	(−0.65,1.01)	-	[1,8.66]
		NDVI	$y=a\times x^{b}+c$	7.24	1.65	0.28	(4.95,9.54)	(0.4,2.9)	(−0.88,1.45)	[0,0.87]
		CI_{Green}	$y=a\times x+b$	1.54	1.22	-	(1.08,2)	(0.43,2.02)	-	[0,3.1]
	TOA Reflectance	SR	$y=a\times x+b$	0.52	0.09	-	(0.38,0.65)	(−0.79,0.97)	-	[1,11.47]
		NDVI	$y=a\times b^{x}$	0.24	41.13	-	(-0.02,0.5)	(−16.47,98.74)	-	[0,0.86]
		EVI	$y=a\times b^{x}$	0.54	13.87	-	(0.19,0.89)	(2.65,25.09)	-	[0,0.92]
		EVI2	$y=a\times x^{b}$	9.39	1.12	-	(6.69,12.08)	(0.72,1.51)	-	[0,0.67]
		CI_{Green}	$y=a\times x+b$	1.03	0.33	-	(0.8,1.26)	(−0.39,1.05)	-	[0,5.5]
	Surface Reflectance	SR	$y=a\times x^{b}+c$	71.57	0.03	−72.90	(−2265.63,2408.78)	(−0.84,0.9)	(−2413.3,2267.5)	[1.92,32.16]
		NDVI	$y=a\times b^{x}$	0.13	54.08	-	(−0.04,0.31)	(−30.08,138.25)	-	[0,0.95]
		EVI	$y=a\times b^{x}$	0.49	31.90	-	(0.17,0.8)	(−0.45,64.25)	-	[0,0.73]
		EVI2	$y=a\times x^{b}$	10.38	1.69	-	(7.26,13.5)	(1.14,2.24)	-	[0,0.72]
		CI_{Green}	$y=a\times x^{b}+c$	14.83	0.14	−15.23	(−87,116.65)	(−0.64,0.92)	(−118.39,87.94)	[1.22,14.12]

Table S4. *Cont.*

		Index	Equation	Coefficient			95% Confidence Interval			Reasonable VI Range
				a	b	c	a	b	c	
**Cotton**	Radance	SR	$y=a\times x^b+c$	2.91	0.54	−2.31	(−1.46,7.28)	(0.02,1.06)	(−6.95,2.34)	[0.65,6.93]
		NDVI	$y=a\times x+b$	6.02	0.45	-	(5.3,6.73)	(0.15,0.74)	-	[0,0.92]
		CI_{Green}	$y=a\times x+b$	1.74	1.05	-	(1.51,1.97)	(0.78,1.32)	-	[0,2.84]
	TOA Reflectance	SR	$y=a\times \log(x)+b$	2.62	−0.85	-	(2.35,2.89)	(−1.19,−0.5)	-	[1.38,13.68]
		NDVI	$y=a\times x^b+c$	8.21	2.14	−0.03	(6.9,9.52)	(1.23,3.05)	(−0.72,0.66)	[0.07,0.87]
		EVI	$y=a\times x^b+c$	5.51	1.26	−0.35	(4.53,6.48)	(0.62,1.89)	(−1.43,0.74)	[0.11,1]
		EVI2	$y=a\times x^b$	9.83	1.69	-	(8.1,11.57)	(1.4,1.98)	-	[0,0.75]
		CI_{Green}	$y=a\times x+b$	1.04	0.04	-	(0.93,1.15)	(−0.24,0.32)	-	[0,5.73]
	Surface Reflectance	SR	$y=a\times x^b+c$	2684.51	0.0008	−2685.45	(−1.59 × 10^6, 1.59 × 10^6)	(−0.47,0.47)	(−1.60 × 10^6, 1.59 × 10^6)	[1.55,25.37]
		NDVI	$y=a^x+b$	8.12	−1.34	-	(7.31,8.94)	(−1.64,−1.04)	-	[0.14,0.95]
		EVI	$y=a\times x^b+c$	6.93	1.21	−0.46	(6.07,7.79)	(0.52,1.9)	-	[0.11,0.94]
		EVI2	$y=a\times x^b$	7.44	1.50	-	(6.43,8.44)	(1.22,1.77)	-	[0,0.87]
		CI_{Green}	$y=a\times x^b+c$	1.58	0.62	−1.10	(−0.49,3.64)	(0.18,1.07)	(−3.37,1.18)	[0.56,11.24]
**Pasture**	Radiance	SR	$y=(x+a)^b$	0.17	0.73	-	(−0.11,0.45)	(0.68,0.78)	-	[1,11.47]
		NDVI	$y=a^x+b$	5.92	−0.02	-	(5.07,6.78)	(-0.22,0.18)	-	[0,1]
		CI_{Green}	$y=a\times x^b+c$	0.95	1.28	0.96	(0.57,1.33)	(0.85,1.71)	(0.7,1.22)	[0,3.67]
	TOA Reflectance	SR	$y=(x+a)^b$	−1.11	0.63	-	(−1.33,−0.88)	(0.59,0.66)	-	[1.11,18.33]
		NDVI	$y=a\times x^b$	4.47	1.33	-	(3.99,4.94)	(1.1,1.55)	-	[0,1]
		EVI	$y=a^x+b$	4.62	−0.57	-	(4.26,4.99)	(−0.75,−0.4)	-	[0,1]
		EVI2	$y=a^{(x+b)}$	24.23	−0.23	-	(15.11,33.35)	(−0.28,−0.19)	-	[0,0.79]
		CI_{Green}	$y=a\times x+b$	0.68	0.41	-	(0.59,0.76)	(0.18,0.64)	-	[0,8.26]
	Surface Reflectance	SR	$y=(x+a)^b$	−1.27	0.53	-	(−1.52,−1.03)	(0.51,0.56)	-	[1.27,29.97]
		NDVI	$y=a^x+b$	5.62	−0.92	-	(5.07,6.17)	(−1.14,−0.7)	-	[0,1]
		EVI	$y=a^x+b$	6.42	−0.62	-	(5.8,7.03)	(−0.8,−0.44)	-	[0,1]
		EVI2	$y=a^{(x+b)}$	17.45	−0.26	-	(11.49,23.42)	(−0.31,−0.21)	-	[0,0.88]
		CI_{Green}	$y=x^a+b$	0.68	−0.41	-	(0.64,0.72)	(−0.59,−0.23)	-	[0.27,15.36]

Table S5. Goodness-Of-Fit (GOF) metrics of the global LAI-VI relationships ($LAI = f(VI)$) for major crops based on three levels of radiometric/atmospheric corrections.

		Index	Regression			Cross Validation			Median and Percentiles of Absolute Residual				
			R^2	RMSE	MAE	R^2	RMSE	MAE	Median	5%	25%	75%	95%
**Overall**	Radiance	SR	0.50	1.12	0.89	0.49 (0.37,0.57)	1.12 (1,1.23)	0.89 (0.8,0.98)	-	-	-	-	-
		NDVI	0.49	1.12	0.90	0.49 (0.38,0.59)	1.12 (1.02,1.22)	0.89 (0.8,0.97)	-	-	-	-	-
		CI_{Green}	0.45	1.18	0.94	0.44 (0.28,0.55)	1.18 (1.07,1.31)	0.93 (0.84,1.04)	-	-	-	-	-
	TOA Reflectance	SR	0.55	1.09	0.86	0.54 (0.44,0.62)	1.08 (0.99,1.18)	0.86 (0.77,0.94)	-	-	-	-	-
		NDVI	0.55	1.09	0.86	0.54 (0.4,0.63)	1.09 (0.99,1.21)	0.86 (0.75,0.97)	-	-	-	-	-
		EVI	0.54	1.10	0.88	0.53 (0.42,0.61)	1.1 (1,1.21)	0.88 (0.8,0.96)	-	-	-	-	-
		EVI2	0.51	1.13	0.92	0.53 (0.43,0.62)	1.1 (1.01,1.19)	0.88 (0.8,0.97)	-	-	-	-	-
		CI_{Green}	0.53	1.11	0.91	0.52 (0.42,0.61)	1.11 (0.98,1.21)	0.91 (0.81,1)	-	-	-	-	-
	Surface Reflectance	SR	0.54	1.10	0.87	0.53 (0.43,0.62)	1.1 (1,1.2)	0.87 (0.78,0.95)	0.71	0.06	0.32	1.31	2.21
		NDVI	0.53	1.11	0.87	0.53 (0.42,0.62)	1.11 (1.02,1.21)	0.87 (0.8,0.95)	0.69	0.06	0.32	1.32	2.22
		EVI	0.52	1.12	0.90	0.52 (0.43,0.62)	1.12 (1.02,1.22)	0.9 (0.82,0.99)	0.73	0.07	0.36	1.34	2.17
		EVI2	0.54	1.10	0.87	0.52 (0.43,0.61)	1.12 (1.02,1.23)	0.87 (0.79,0.96)	0.72	0.06	0.37	1.30	2.12
		CI_{Green}	0.53	1.11	0.87	0.53 (0.42,0.62)	1.11 (1,1.22)	0.88 (0.79,0.98)	0.71	0.06	0.31	1.29	2.24
**Rowcrop**	Radiance	SR	0.52	1.11	0.87	0.51 (0.36,0.64)	1.12 (0.97,1.22)	0.88 (0.76,0.98)	-	-	-	-	-
		NDVI	0.52	1.10	0.88	0.52 (0.36,0.63)	1.11 (0.98,1.21)	0.88 (0.77,0.98)	-	-	-	-	-
		CI_{Green}	0.48	1.16	0.92	0.47 (0.29,0.58)	1.17 (1.03,1.32)	0.93 (0.81,1.04)	-	-	-	-	-
	TOA Reflectance	SR	0.57	1.08	0.85	0.56 (0.46,0.65)	1.08 (0.97,1.18)	0.86 (0.76,0.95)	-	-	-	-	-
		NDVI	0.56	1.09	0.85	0.55 (0.41,0.65)	1.09 (0.99,1.2)	0.85 (0.76,0.94)	-	-	-	-	-
		EVI	0.55	1.10	0.87	0.54 (0.44,0.62)	1.1 (0.99,1.22)	0.87 (0.78,0.97)	-	-	-	-	-
		EVI2	0.52	1.13	0.91	0.55 (0.43,0.66)	1.1 (0.96,1.22)	0.87 (0.75,0.98)	-	-	-	-	-
		CI_{Green}	0.55	1.09	0.90	0.55 (0.44,0.63)	1.09 (1,1.19)	0.9 (0.81,0.98)	-	-	-	-	-
	Surface Reflectance	SR	0.54	1.11	0.88	0.54 (0.42,0.63)	1.11 (1,1.22)	0.88 (0.79,0.98)	0.72	0.06	0.32	1.34	2.15
		NDVI	0.55	1.10	0.87	0.54 (0.41,0.64)	1.1 (0.99,1.23)	0.88 (0.77,1)	0.74	0.06	0.30	1.32	2.11
		EVI	0.53	1.12	0.89	0.52 (0.42,0.6)	1.12 (1.01,1.22)	0.9 (0.8,1)	0.73	0.06	0.36	1.31	2.21
		EVI2	0.55	1.10	0.87	0.55 (0.43,0.65)	1.1 (0.98,1.2)	0.87 (0.78,0.98)	0.70	0.07	0.35	1.25	2.17
		CI_{Green}	0.55	1.09	0.88	0.55 (0.47,0.64)	1.1 (0.98,1.21)	0.89 (0.78,0.98)	0.77	0.07	0.36	1.28	2.12

Table S5. *Cont.*

		Index	Regression			Cross Validation			Median and Percentiles of Absolute Residual				
			R^2	RMSE	MAE	R^2	RMSE	MAE	Median	5%	25%	75%	95%
**Maize**	Radiance	SR	0.51	1.04	0.83	0.49 (0.09,0.69)	1.04 (0.84,1.24)	0.84 (0.68,0.99)	-	-	-	-	-
		NDVI	0.51	1.03	0.84	0.5 (0.23,0.67)	1.04 (0.84,1.22)	0.85 (0.69,1.03)	-	-	-	-	-
		CI_{Green}	0.39	1.15	0.96	0.37 (0.1,0.52)	1.16 (0.96,1.37)	0.97 (0.79,1.17)	-	-	-	-	-
	TOA Reflectance	SR	0.58	0.98	0.80	0.56 (0.28,0.72)	0.99 (0.78,1.22)	0.81 (0.65,0.97)	-	-	-	-	-
		NDVI	0.56	1.00	0.79	0.55 (0.3,0.72)	1.01 (0.83,1.22)	0.79 (0.65,0.94)	-	-	-	-	-
		EVI	0.63	0.93	0.72	0.62 (0.39,0.79)	0.93 (0.74,1.11)	0.73 (0.6,0.9)	-	-	-	-	-
		EVI2	0.49	1.08	0.87	0.62 (0.31,0.77)	0.93 (0.68,1.11)	0.73 (0.54,0.87)	-	-	-	-	-
		CI_{Green}	0.52	1.05	0.82	0.51 (0.23,0.71)	1.06 (0.87,1.27)	0.83 (0.68,1.01)	-	-	-	-	-
	Surface Reflectance	SR	0.59	0.98	0.79	0.57 (0.35,0.76)	0.98 (0.8,1.19)	0.79 (0.66,0.97)	0.68	0.09	0.36	1.13	1.95
		NDVI	0.56	1.01	0.78	0.55 (0.23,0.74)	1.01 (0.8,1.2)	0.79 (0.63,0.96)	0.60	0.07	0.29	1.19	2.06
		EVI	0.54	1.03	0.81	0.54 (0.23,0.75)	1.03 (0.8,1.27)	0.81 (0.64,0.99)	0.72	0.07	0.31	1.16	2.10
		EVI2	0.62	0.94	0.73	0.6 (0.37,0.75)	0.94 (0.75,1.14)	0.74 (0.58,0.9)	0.61	0.05	0.30	1.02	1.86
		CI_{Green}	0.58	0.99	0.77	0.56 (0.31,0.75)	1 (0.79,1.17)	0.78 (0.6,0.94)	0.60	0.07	0.28	1.13	1.99
**Soybean**	Radiance	SR	0.52	0.96	0.74	0.43 (−0.33,0.78)	1.01 (0.64,1.49)	0.79 (0.51,1.13)	-	-	-	-	-
		NDVI	0.59	0.88	0.63	0.54 (−0.08,0.85)	0.9 (0.52,1.26)	0.65 (0.37,1)	-	-	-	-	-
		CI_{Green}	0.61	0.86	0.60	0.58 (0.11,0.88)	0.86 (0.44,1.28)	0.62 (0.3,1.01)	-	-	-	-	-
	TOA Reflectance	SR	0.68	0.78	0.55	0.66 (−0.08,0.9)	0.79 (0.42,1.17)	0.57 (0.33,0.89)	-	-	-	-	-
		NDVI	0.67	0.79	0.56	0.63 (0.06,0.91)	0.8 (0.44,1.13)	0.58 (0.3,0.88)	-	-	-	-	-
		EVI	0.73	0.72	0.50	0.69 (−0.08,0.93)	0.74 (0.33,1.15)	0.52 (0.25,0.83)	-	-	-	-	-
		EVI2	0.72	0.73	0.52	0.69 (−0.12,0.94)	0.73 (0.32,1.11)	0.51 (0.26,0.8)	-	-	-	-	-
		CI_{Green}	0.69	0.77	0.55	0.65 (0.06,0.89)	0.79 (0.42,1.17)	0.57 (0.31,0.9)	-	-	-	-	-
	Surface Reflectance	SR	0.67	0.80	0.59	0.62 (0.13,0.86)	0.83 (0.46,1.15)	0.65 (0.37,0.98)	0.42	0.03	0.16	0.74	1.74
		NDVI	0.68	0.79	0.56	0.65 (0.1,0.9)	0.79 (0.42,1.15)	0.57 (0.33,0.89)	0.42	0.02	0.13	0.71	1.77
		EVI	0.75	0.69	0.48	0.72 (0.09,0.94)	0.7 (0.38,1.08)	0.5 (0.28,0.8)	0.35	0.01	0.11	0.65	1.44
		EVI2	0.73	0.72	0.51	0.69 (0.22,0.92)	0.73 (0.38,1.03)	0.53 (0.28,0.77)	0.33	0.02	0.14	0.72	1.50
		CI_{Green}	0.67	0.80	0.54	0.64 (0.14,0.89)	0.81 (0.4,1.16)	0.56 (0.31,0.9)	0.33	0.02	0.13	0.74	1.88

Table S5. *Cont.*

		Index	Regression			Cross Validation			Median and Percentiles of Absolute Residual				
			R^2	RMSE	MAE	R^2	RMSE	MAE	Median	5%	25%	75%	95%
**Wheat**	Radiance	SR	0.61	0.93	0.93	0.42 (0.07,0.66)	1.19 (0.93,1.51)	0.95 (0.71,1.21)	-	-	-	-	-
		NDVI	0.59	0.95	0.94	0.42 (0.1,0.66)	1.19 (0.92,1.44)	0.96 (0.73,1.18)	-	-	-	-	-
		CI_{Green}	0.50	1.08	1.00	0.43 (0.11,0.62)	1.21 (1.01,1.44)	1.03 (0.86,1.28)	-	-	-	-	-
	TOA Reflectance	SR	0.55	1.05	1.01	0.41 (0.05,0.57)	1.24 (1.01,1.46)	1.03 (0.81,1.24)	-	-	-	-	-
		NDVI	0.52	1.09	1.04	0.4 (−0.02,0.57)	1.25 (1.05,1.55)	1.06 (0.85,1.28)	-	-	-	-	-
		EVI	0.59	1.01	0.98	0.44 (0.13,0.63)	1.21 (1.01,1.41)	1 (0.81,1.18)	-	-	-	-	-
		EVI2	0.58	1.01	0.97	0.44 (−0.01,0.64)	1.21 (0.98,1.53)	1 (0.79,1.27)	-	-	-	-	-
		CI_{Green}	0.45	1.16	1.07	0.4 (0.17,0.55)	1.26 (1.04,1.5)	1.09 (0.86,1.33)	-	-	-	-	-
	Surface Reflectance	SR	0.60	0.99	0.97	0.43 (−0.02,0.64)	1.22 (0.93,1.48)	0.99 (0.77,1.23)	0.88	0.09	0.40	1.36	2.48
		NDVI	0.57	1.02	1.00	0.43 (0.1,0.6)	1.22 (0.95,1.43)	1.01 (0.81,1.21)	0.92	0.12	0.49	1.44	2.24
		EVI	0.62	0.97	0.93	0.5 (0.21,0.68)	1.14 (0.91,1.35)	0.94 (0.72,1.16)	0.86	0.10	0.43	1.33	2.08
		EVI2	0.62	0.97	0.94	0.5 (0.21,0.66)	1.15 (0.92,1.38)	0.95 (0.75,1.15)	0.84	0.11	0.43	1.39	2.08
		CI_{Green}	0.51	1.10	1.02	0.42 (0.11,0.61)	1.23 (1,1.48)	1.04 (0.81,1.25)	0.94	0.12	0.49	1.46	2.15
**Rice**	Radiance	SR	0.74	0.97	0.89	0.51 (−1.99,0.9)	1.21 (0.62,2.05)	0.93 (0.49,1.52)	-	-	-	-	-
		NDVI	0.74	0.98	0.86	0.4 (−2.46,0.9)	1.36 (0.57,2.3)	1 (0.45,1.64)	-	-	-	-	-
		CI_{Green}	0.66	0.95	0.86	0.39 (−4.74,0.87)	1.16 (0.54,2.02)	0.91 (0.46,1.61)	-	-	-	-	-
	TOA Reflectance	SR	0.71	1.04	0.94	0.5 (−3.12,0.88)	1.25 (0.66,2.06)	0.99 (0.5,1.71)	-	-	-	-	-
		NDVI	0.71	1.03	0.89	0.47 (−4.95,0.92)	1.25 (0.56,2.15)	0.96 (0.44,1.56)	-	-	-	-	-
		EVI	0.74	0.97	0.86	0.48 (−6.18,0.92)	1.22 (0.62,2.08)	0.96 (0.53,1.57)	-	-	-	-	-
		EVI2	0.75	0.95	0.81	0.57 (−0.67,0.93)	1.15 (0.5,1.95)	0.87 (0.39,1.46)	-	-	-	-	-
		CI_{Green}	0.76	0.94	0.82	0.58 (−1.4,0.93)	1.12 (0.54,1.82)	0.86 (0.44,1.48)	-	-	-	-	-
	Surface Reflectance	SR	0.63	1.18	0.96	0.47 (−0.91,0.88)	1.29 (0.69,1.99)	1.02 (0.54,1.63)	0.89	0.06	0.36	1.41	2.67
		NDVI	0.65	1.14	0.95	0.45 (−5.12,0.89)	1.3 (0.58,2.15)	1.03 (0.48,1.68)	0.85	0.07	0.42	1.40	2.27
		EVI	0.73	1.00	0.83	0.53 (−5.88,0.91)	1.18 (0.62,2.01)	0.91 (0.5,1.59)	0.75	0.01	0.45	1.11	2.63
		EVI2	0.74	0.98	0.78	0.54 (−3.14,0.94)	1.17 (0.54,2.03)	0.89 (0.44,1.56)	0.66	0.02	0.33	0.92	2.63
		CI_{Green}	0.69	1.09	0.87	0.52 (−1.1,0.89)	1.21 (0.59,1.87)	0.94 (0.48,1.46)	0.77	0.11	0.33	1.30	2.19

Table S5. *Cont.*

		Index	Regression			Cross Validation			Median and Percentiles of Absolute Residual				
			R^2	RMSE	MAE	R^2	RMSE	MAE	Median	5%	25%	75%	95%
Cotton	Radiance	SR	0.78	0.81	0.63	0.72 (−0.61,0.92)	0.85 (0.51,1.21)	0.68 (0.4,0.97)	-	-	-	-	-
		NDVI	0.77	0.82	0.68	0.72 (−0.02,0.9)	0.85 (0.57,1.23)	0.7 (0.47,1.03)	-	-	-	-	-
		CI_{Green}	0.74	0.87	0.73	0.69 (−0.01,0.89)	0.89 (0.6,1.22)	0.75 (0.51,1.01)	-	-	-	-	-
	TOA Reflectance	SR	0.78	0.83	0.66	0.74 (−0.01,0.93)	0.86 (0.53,1.26)	0.68 (0.41,1.04)	-	-	-	-	-
		NDVI	0.74	0.90	0.67	0.68 (−0.08,0.89)	0.93 (0.59,1.31)	0.72 (0.48,0.97)	-	-	-	-	-
		EVI	0.73	0.92	0.67	0.67 (−0.2,0.91)	0.95 (0.55,1.48)	0.72 (0.41,1.14)	-	-	-	-	-
		EVI2	0.75	0.88	0.66	0.66 (−0.58,0.91)	0.96 (0.46,1.49)	0.72 (0.39,1.11)	-	-	-	-	-
		CI_{Green}	0.79	0.80	0.66	0.76 (0.4,0.9)	0.82 (0.6,1.06)	0.67 (0.45,0.91)	-	-	-	-	-
	Surface Reflectance	SR	0.68	1.00	0.73	0.64 (−0.39,0.9)	1.01 (0.5,1.46)	0.74 (0.35,1.09)	0.50	0.03	0.19	1.22	1.98
		NDVI	0.69	0.98	0.72	0.62 (−0.91,0.91)	1 (0.58,1.44)	0.74 (0.46,1.11)	0.51	0.04	0.21	1.10	2.09
		EVI	0.70	0.97	0.70	0.63 (−0.48,0.89)	1.02 (0.57,1.54)	0.76 (0.44,1.16)	0.61	0.05	0.40	1.27	2.01
		EVI2	0.67	1.01	0.76	0.59 (−0.63,0.88)	1.07 (0.58,1.73)	0.8 (0.47,1.31)	0.54	0.09	0.30	1.09	2.20
		CI_{Green}	0.72	0.93	0.67	0.66 (−0.33,0.9)	0.96 (0.54,1.44)	0.72 (0.4,1.09)	0.43	0.02	0.23	0.96	1.83
Pasture	Radiance	SR	0.49	1.00	0.82	0.46 (−0.25,0.63)	1.02 (0.84,1.2)	0.84 (0.69,1.04)	-	-	-	-	-
		NDVI	0.46	1.03	0.85	0.44 (0.09,0.62)	1.04 (0.7,1.25)	0.86 (0.56,1.05)	-	-	-	-	-
		CI_{Green}	0.47	1.05	0.87	0.43 (0.06,0.6)	1.08 (0.84,1.3)	0.89 (0.71,1.07)	-	-	-	-	-
	TOA Reflectance	SR	0.50	1.01	0.80	0.49 (0.2,0.74)	1.02 (0.73,1.27)	0.82 (0.6,1.06)	-	-	-	-	-
		NDVI	0.48	1.04	0.83	0.45 (0.14,0.63)	1.05 (0.79,1.35)	0.84 (0.63,1.06)	-	-	-	-	-
		EVI	0.52	0.99	0.81	0.5 (−0.01,0.7)	1.01 (0.82,1.24)	0.82 (0.63,1)	-	-	-	-	-
		EVI2	0.51	1.00	0.80	0.5 (0.09,0.73)	1.01 (0.8,1.29)	0.82 (0.66,1.06)	-	-	-	-	-
		CI_{Green}	0.49	1.02	0.85	0.47 (0.19,0.62)	1.04 (0.82,1.24)	0.86 (0.69,1.03)	-	-	-	-	-
	Surface Reflectance	SR	0.50	1.02	0.80	0.48 (−0.01,0.77)	1.03 (0.74,1.28)	0.82 (0.6,1.02)	0.68	0.08	0.32	1.12	2.14
		NDVI	0.49	1.02	0.82	0.46 (0.07,0.66)	1.04 (0.83,1.25)	0.83 (0.63,1.01)	0.70	0.08	0.31	1.20	2.10
		EVI	0.52	1.00	0.81	0.49 (0.1,0.68)	1.01 (0.79,1.26)	0.83 (0.64,1.02)	0.71	0.09	0.37	1.11	1.99
		EVI2	0.51	1.00	0.80	0.49 (−0.12,0.7)	1.01 (0.8,1.35)	0.82 (0.66,1.1)	0.69	0.07	0.38	1.09	2.11
		CI_{Green}	0.47	1.04	0.84	0.44 (−0.01,0.66)	1.06 (0.86,1.29)	0.86 (0.7,1.05)	0.70	0.08	0.33	1.16	2.12

Table S6. Best-fit functions for the LAI-VI relationships ($LAI = f(VI)$) for major crops based on surface reflectance using the dataset with a complete LAI data range.

	**Index**	**Equation**	**Coefficient**			**95% Confidence Interval**		
			**a**	**b**	**c**	**a**	**b**	**c**
Overall	SR	$y = a \times \log(b \times x + c)$	1.28	1.87	−1.71	(1.13,1.42)	(1.24,2.5)	(−2.75,−0.66)
	NDVI	$y = a \times x^b$	5.22	1.69	-	(5.03,5.41)	(1.56,1.82)	-
	EVI	$y = a \times x + b$	5.55	−0.11	-	(5.25,5.84)	(−0.27,0.06)	-
	EVI2	$y = a \times x + b$	6.11	−0.17	-	(5.79,6.43)	(−0.33,0)	-
	CI_{Green}	$y = a \times b^x + c$	−5.93	0.71	4.45	(−6.24,−5.61)	(0.68,0.75)	(4.23,4.67)
Rowcrop	SR	$y = a \times \log(bx + c)$	1.55	1.12	−0.69	(1.34,1.76)	(0.72,1.51)	(−1.38,0.01)
	NDVI	$y = a \times x^b + c$	5.76	1.70	−0.30	(5.44,6.09)	(1.37,2.03)	(−0.72,0.11)
	EVI	$y = a \times b^x + c$	−9.36	0.34	8.17	(−11.89,−6.84)	(0.15,0.53)	(5.29,11.05)
	EVI2	$y = a \times \log(x) + b$	2.35	4.64	-	(2.21,2.49)	(4.5,4.78)	-
	CI_{Green}	$y = a \times \log(x) + b$	1.97	−0.02	-	(1.86,2.07)	(−0.18,0.14)	-
Maize	SR	$y = a \times \log(x) + b$	2.17	−0.91	-	(2.02,2.31)	(−1.2,−0.61)	-
	NDVI	$y = a \times x^b$	6.61	2.31	-	(6.15,7.07)	(2.01,2.61)	-
	EVI	$y = a \times e^x + b$	5.07	−5.55	-	(4.71,5.43)	(−6.18,−4.92)	-
	EVI2	$y = a \times e^x + b$	5.71	−6.24	-	(5.34,6.08)	(−6.85,−5.62)	-
	CI_{Green}	$y = a \times x^b + c$	4.25	0.35	−4.15	(0.58,7.91)	(0.14,0.55)	(−8.02,−0.29)
Soybean	SR	$y = a \times \log(b \times x + c)$	0.86	2.23	−3.00	(0.61,1.12)	(−0.04,4.51)	(−7.26,1.26)
	NDVI	$y = a \times x + b$	4.73	−1.22	-	(4,5.47)	(−1.8,−0.64)	-
	EVI	$y = a \times \log(x) + b$	1.82	3.24	-	(1.52,2.11)	(3.04,3.43)	-
	EVI2	$y = a \times \log(x) + b$	1.80	3.33	-	(1.51,2.1)	(3.11,3.54)	-
	CI_{Green}	$y = \log(a \times x + b)$	2.21	−1.74	-	(1.83,2.6)	(−2.5,−0.98)	-
Wheat	SR	$y = a \times \log(b \times x + c)$	1.51	2.59	−2.85	(1.18,1.84)	(0.9,4.28)	(−5.63,−0.07)
	NDVI	$y = a \times x^b$	7.24	1.72	-	(6.66,7.82)	(1.49,1.96)	-
	EVI	$y = a \times x + b$	8.99	−0.95	-	(8.15,9.82)	(−1.36,−0.54)	-
	EVI2	$y = a \times x + b$	10.04	−1.11	-	(9.08,11)	(−1.55,−0.68)	-
	CI_{Green}	$y = a \times b^x + c$	−9.52	0.64	5.66	(−10.86,−8.19)	(0.57,0.71)	(5.18,6.14)

Table S6. *Cont.*

	Index	Equation	Coefficient			95% Confidence Interval		
			a	b	c	a	b	c
Rice	SR	$y=a\times\log(x)+b$	2.27	−1.61	-	(1.64,2.9)	(−3,−0.22)	-
	NDVI	$y=a\times x^b$	6.83	2.93	-	(5.07,8.59)	(1.58,4.29)	-
	EVI	$y=a\times x+b$	7.96	−0.41	-	(6.07,9.86)	(−1.39,0.56)	-
	EVI2	$y=a\times x+b$	8.43	−0.43	-	(6.44,10.41)	(−1.4,0.54)	-
	CI_{Green}	$y=a\times\log(x)+b$	2.36	−0.13	-	(1.8,2.92)	(−1.04,0.78)	-
Cotton	SR	$y=a\times\log(x)+b$	2.06	−0.50	-	(1.84,2.27)	(−0.84,−0.16)	-
	NDVI	$y=a\times x^b$	6.00	1.70	-	(5.4,6.59)	(1.41,1.99)	-
	EVI	$y=a\times e^x+b$	4.57	−4.89	-	(4.19,4.96)	(−5.52,−4.26)	-
	EVI2	$y=a\times x+b$	6.75	−0.50	-	(5.95,7.56)	(−0.88,−0.12)	-
	CI_{Green}	$y=x^a+b$	0.77	−0.30	-	(0.74,0.81)	(−0.53,−0.07)	-
Pasture	SR	$y=(x+a)^b$	−1.01	0.53	-	(−1.33,−0.7)	(0.5,0.56)	-
	NDVI	$y=a\times b^x$	0.41	12.71	-	(0.29,0.53)	(7.86,17.56)	-
	EVI	$y=a\times b^x$	0.63	9.47	-	(0.5,0.76)	(6.71,12.23)	-
	EVI2	$y=a\times b^x$	0.61	11.90	-	(0.49,0.74)	(8.09,15.7)	-
	CI_{Green}	$y=a\times(x+b)^c$	0.98	−0.37	0.66	(0.38,1.58)	(−1.41,0.67)	(0.38,0.94)

Table S7. GOF metrics of the global LAI-VI relationships ($LAI=f(VI)$) for major crops based on three levels of radiometric/atmospheric corrections.

	Index	Regression			Cross Validation			Median and Percentiles of Absolute Residual				
		R^2	RMSE	MAE	R^2	RMSE	MAE	Median	5%	25%	75%	95%
Overall	SR	0.50	1.35	1.07	0.48	1.38	1.07	0.69	0.03	0.29	1.24	1.98
	NDVI	0.51	1.34	1.05	0.49	1.37	1.05	0.65	0.02	0.26	1.33	2.11
	EVI	0.43	1.45	1.12	0.43	1.45	1.12	1.13	0.22	0.63	1.67	3.04
	EVI2	0.43	1.44	1.11	0.43	1.44	1.11	0.95	0.07	0.42	1.67	2.98
	CI_{Green}	0.52	1.33	1.05	0.49	1.36	1.05	0.90	0.10	0.36	1.50	2.61
Row crop	SR	0.46	1.44	1.09	0.46	1.44	1.09	0.90	0.08	0.36	1.50	2.59
	NDVI	0.48	1.41	1.07	0.46	1.43	1.09	0.94	0.05	0.37	1.69	2.73
	EVI	0.43	1.47	1.11	0.43	1.47	1.11	0.84	0.13	0.39	1.71	3.62
	EVI2	0.43	1.47	1.13	0.43	1.47	1.13	0.82	0.05	0.36	1.75	3.45
	CI_{Green}	0.46	1.44	1.09	0.46	1.44	1.09	0.64	0.03	0.24	1.53	3.17

Table S7. *Cont.*

	Index	Regression			Cross Validation			Median and Percentiles of Absolute Residual				
		R^2	RMSE	MAE	R^2	RMSE	MAE	Median	5%	25%	75%	95%
Maize	SR	0.66	1.21	0.94	0.60	1.31	0.95	0.57	0.03	0.21	1.52	3.12
	NDVI	0.64	1.24	0.96	0.59	1.33	0.97	0.86	0.00	0.39	1.46	2.76
	EVI	0.62	1.27	1.01	0.56	1.37	1.02	0.58	0.11	0.30	0.98	2.24
	EVI2	0.69	1.16	0.95	0.60	1.32	0.96	0.64	0.12	0.36	1.01	2.13
	CI_{Green}	0.66	1.20	0.97	0.58	1.35	0.97	0.48	0.11	0.26	0.94	2.51
Soybean	SR	0.42	1.08	0.83	0.39	1.09	0.85	0.61	0.12	0.37	1.08	2.02
	NDVI	0.42	1.07	0.83	0.40	1.09	0.85	0.46	0.04	0.30	0.93	2.10
	EVI	0.43	1.07	0.83	0.41	1.08	0.84	0.67	0.08	0.34	1.16	2.17
	EVI2	0.42	1.08	0.84	0.40	1.09	0.85	0.70	0.05	0.34	1.20	2.13
	CI_{Green}	0.41	1.08	0.83	0.39	1.10	0.84	0.72	0.07	0.42	1.09	2.04
Wheat	SR	0.48	1.48	1.17	0.45	1.51	1.19	0.70	0.04	0.42	1.10	2.02
	NDVI	0.50	1.45	1.13	0.48	1.46	1.15	0.73	0.07	0.39	1.20	2.17
	EVI	0.60	1.29	1.03	0.59	1.30	1.03	0.69	0.03	0.29	1.24	1.98
	EVI2	0.59	1.31	1.04	0.58	1.33	1.05	0.65	0.02	0.26	1.33	2.11
	CI_{Green}	0.56	1.36	1.07	0.55	1.37	1.09	1.13	0.22	0.63	1.67	3.04
Rice	SR	0.44	1.63	1.19	0.35	1.68	1.26	0.95	0.07	0.42	1.67	2.98
	NDVI	0.46	1.61	1.17	0.34	1.68	1.26	0.90	0.10	0.36	1.50	2.61
	EVI	0.58	1.42	0.98	0.51	1.47	1.02	0.90	0.08	0.36	1.50	2.59
	EVI2	0.58	1.41	0.97	0.50	1.46	1.01	0.94	0.05	0.37	1.69	2.73
	CI_{Green}	0.58	1.42	1.06	0.52	1.45	1.11	0.84	0.13	0.39	1.71	3.62
Cotton	SR	0.76	0.93	0.75	0.67	1.04	0.78	0.82	0.05	0.36	1.75	3.45
	NDVI	0.74	0.97	0.77	0.68	1.03	0.81	0.64	0.03	0.24	1.53	3.17
	EVI	0.81	0.82	0.72	0.66	1.05	0.75	0.57	0.03	0.21	1.52	3.12
	EVI2	0.73	0.98	0.78	0.70	1.00	0.79	0.86	0.00	0.39	1.46	2.76
	CI_{Green}	0.78	0.89	0.71	0.70	0.99	0.74	0.58	0.11	0.30	0.98	2.24
Pasture	SR	0.51	1.05	0.83	0.48	1.07	0.84	0.64	0.12	0.36	1.01	2.13
	NDVI	0.50	1.07	0.84	0.47	1.09	0.86	0.48	0.11	0.26	0.94	2.51
	EVI	0.53	1.04	0.84	0.50	1.05	0.84	0.61	0.12	0.37	1.08	2.02
	EVI2	0.52	1.04	0.83	0.49	1.05	0.84	0.46	0.04	0.30	0.93	2.10
	CI_{Green}	0.47	1.10	0.86	0.45	1.11	0.88	0.67	0.08	0.34	1.16	2.17

Table S8. LAI-EVI and LAI-EVI2 relationships ($LAI = f(VI)$) based on data transformation and simple linear regression (SLR) with Theil-Sen estimator. (The complete-range data).

Crop Type	VI	SLR Model	Coefficient (Confidence Interval)		Prediction Model	RMSE (m^2/m^2)	MAE (m^2/m^2)	Quantiles of Absolute Residuals (m^2/m^2)				
			a	b				5%	25%	50%	75%	95%
Overall	EVI	$\sqrt{y} = a\frac{1}{\sqrt[3]{x}} + b$	3.51 (3.35,6.67)	−1.22	$y = \left(a\frac{1}{\sqrt[3]{x}} + b\right)^2$	1.46	1.09	0.07	0.34	0.84	1.6	2.93
	EVI2	$\sqrt{y} = a\frac{1}{\sqrt[3]{x}} + b$	3.73 (3.56,3.89)	−1.32	$y = \left(a\frac{1}{\sqrt[3]{x}} + b\right)^2$	1.45	1.08	0.07	0.33	0.8	1.56	2.98
Row crop	EVI	$\sqrt[3]{y} = a\sqrt[4]{x} + b$	2.81 (2.68,2.95)	−1.02	$y = (a\sqrt[4]{x} + b)^3$	1.52	1.13	0.07	0.32	0.84	1.67	3.24
	EVI2	$\sqrt[3]{y} = a\sqrt[4]{x} + b$	2.97 (2.83,3.11)	−1.11	$y = (a\sqrt[4]{x} + b)^3$	1.52	1.12	0.06	0.32	0.8	1.65	3.32
Maize	EVI	$\sqrt{y} = a\sqrt{x} + b$	3.8 (3.57,4.03)	−1.03	$y = (a\sqrt{x} + b)^2$	1.37	1.01	0.04	0.31	0.74	1.43	2.87
	EVI2	$\sqrt{y} = a\sqrt[3]{x} + b$	5.06 (4.79,5.34)	−2.23	$y = (a\sqrt[3]{x} + b)^2$	1.31	0.94	0.03	0.26	0.66	1.31	2.85
Soybean	EVI	$\sqrt[4]{y} = a\frac{1}{\sqrt{x}} + b$	-0.51 (−0.57,−0.44)	1.88	$y = \left(a\frac{1}{\sqrt{x}} + b\right)^4$	1.07	0.82	0.04	0.24	0.68	1.3	2.04
	EVI2	$\sqrt[3]{y} = a\frac{1}{\sqrt{x}} + b$	−0.69 (−0.74,−0.56)	2.19	$y = \left(a\frac{1}{\sqrt{x}} + b\right)^3$	1.06	0.82	0.05	0.23	0.67	1.31	2
Wheat	EVI	$y^{\frac{3}{5}} = ax + b$	3.73 (3.37,4.07)	0.23	$y = (ax + b)^{\frac{5}{3}}$	1.3	1.04	0.89	0.4	0.88	1.55	2.52
	EVI2	$y^{\frac{3}{5}} = ax^{\frac{3}{5}} + b$	4.82 (4.36,5.28)	−0.87	$y = \left(ax^{\frac{3}{5}} + b\right)^{\frac{5}{3}}$	1.32	1.04	0.1	0.34	0.88	1.46	2.66
Rice	EVI	$\sqrt{y} = ax^{\frac{2}{5}} + b$	3.83 (3.06,4.46)	−1.05	$y = \left(ax^{\frac{2}{5}} + b\right)^2$	1.41	0.97	0.01	1.18	0.65	1.27	3.08
	EVI2	$\sqrt{y} = ax^{\frac{2}{5}} + b$	3.9 (3.14,4.57)	−1.04	$y = \left(ax^{\frac{2}{5}} + b\right)^2$	1.4	0.96	0.03	0.2	0.58	1.24	3.02
Cotton	EVI	$\sqrt[3]{y} = a\frac{1}{\sqrt[3]{x}} + b$	−1.28 (−1.4,−1.16)	3.03	$y = \left(a\frac{1}{\sqrt[3]{x}} + b\right)^3$	0.94	0.73	0.04	0.22	0.66	1.09	1.91
	EVI2	$\sqrt[3]{y} = a\frac{1}{\sqrt[3]{x}} + b$	−1.24 (−1.35,−1.11)	3.02	$y = \left(a\frac{1}{\sqrt[3]{x}} + b\right)^3$	0.97	0.77	0.03	0.28	0.66	1.15	1.99
Pasture	EVI	$\sqrt{y} = ax^{\frac{6}{5}} + b$	1.6 (1.4,1.81)	0.71	$y = \left(ax^{\frac{6}{5}} + b\right)^2$	1.05	0.84	0.08	0.38	0.7	1.12	2.1
	EVI2	$\sqrt{y} = ax^{\frac{4}{3}} + b$	1.79 (1.57,2.03)	0.75	$y = \left(ax^{\frac{4}{3}} + b\right)^2$	1.05	0.84	0.07	0.39	0.69	1.11	2.07

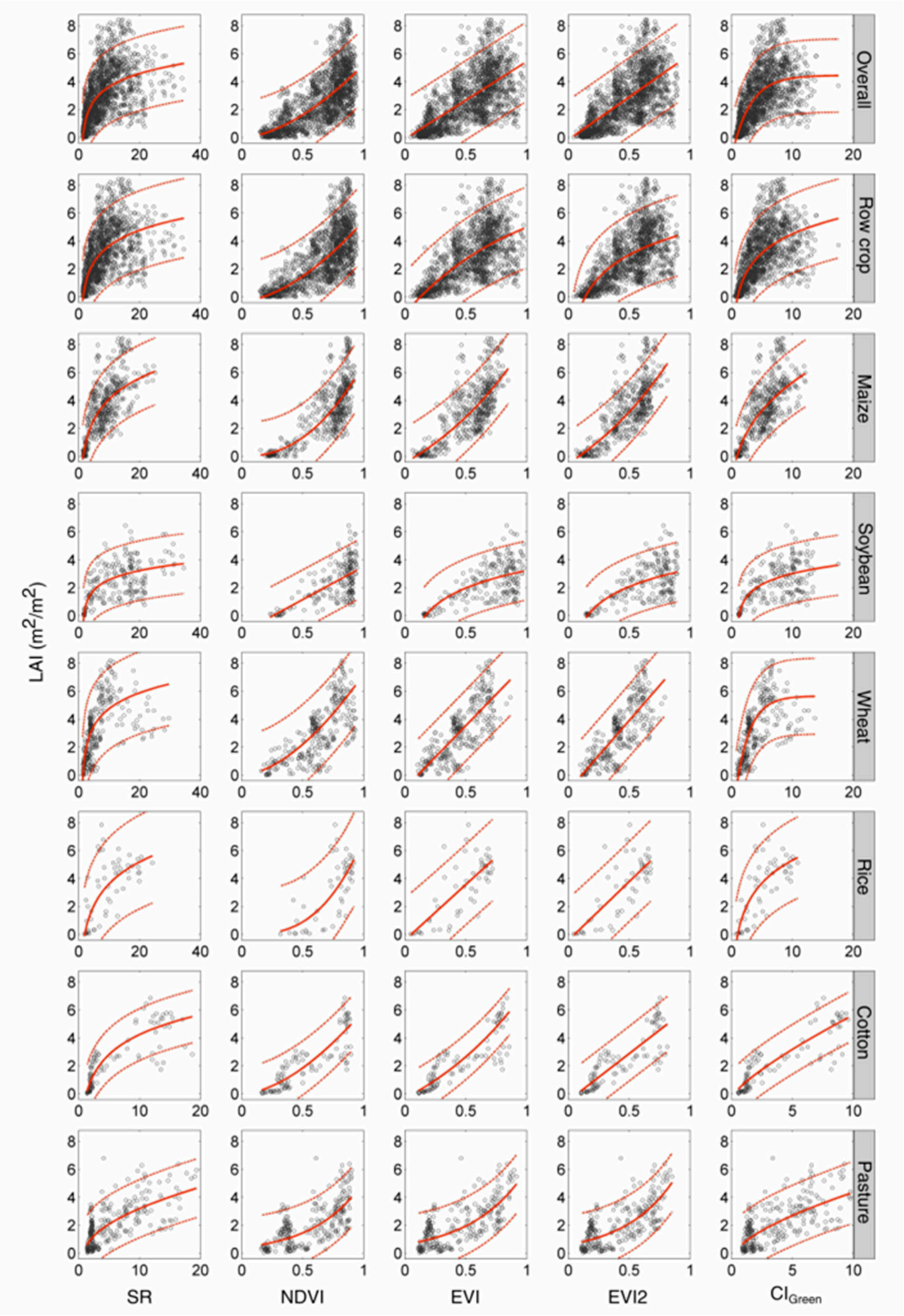

Figure S6. Best-fit functions of global LAI-VI relationships based on in situ LAI and surface reflectance based VIs (SR, NDVI, EVI, EVI2, and CIGreen) for the full-range dataset. Best-fit functions are plotted by solid red lines, and prediction intervals (95%) are presented in dashed red lines.

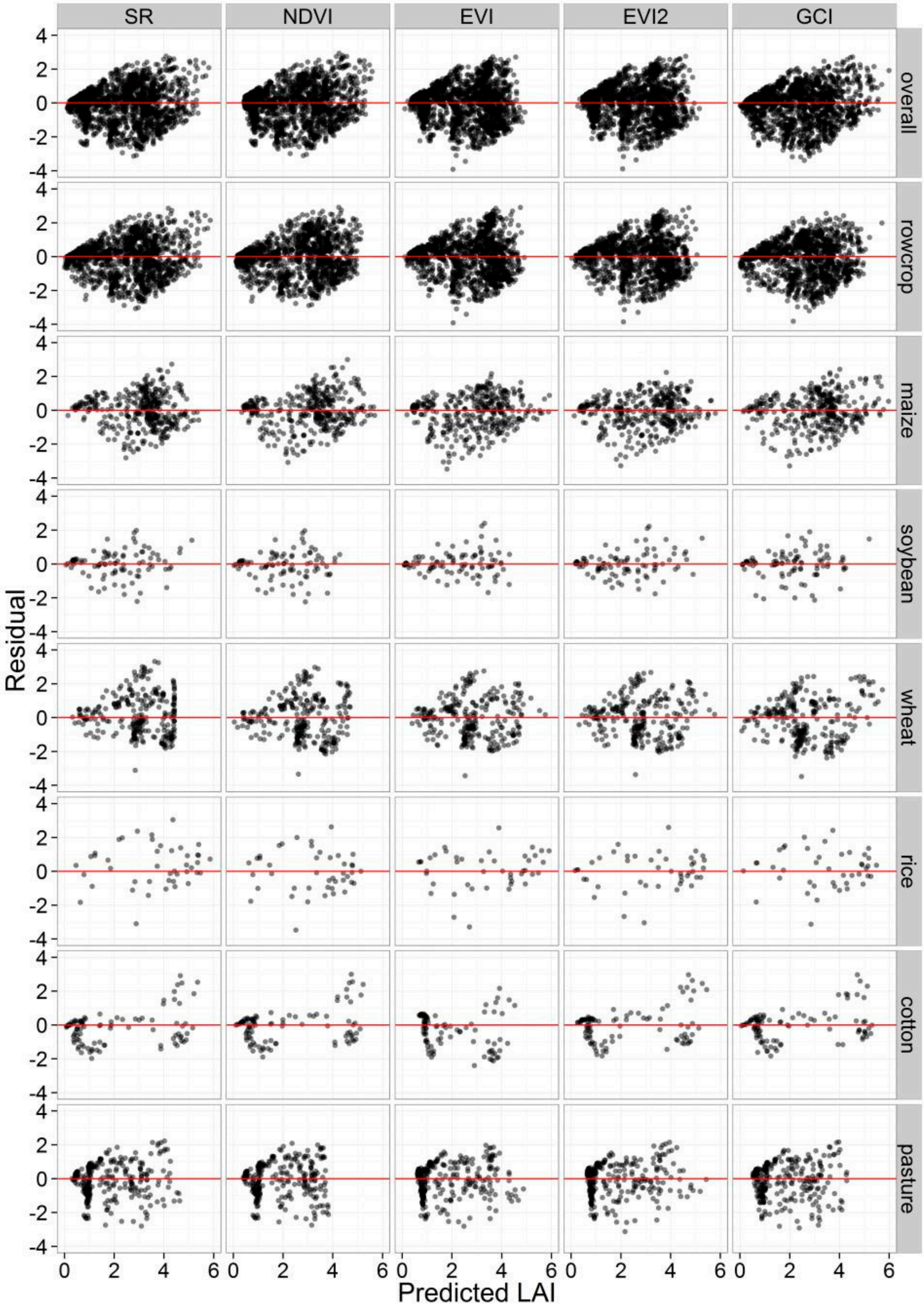

Figure S7. Residuals of the global overall LAI-VI relationships (from surface reflectance) plotted against predicted LAI values. The residual variance shows an unequal pattern across different ranges of LAI values for most of the crop types and VIs.

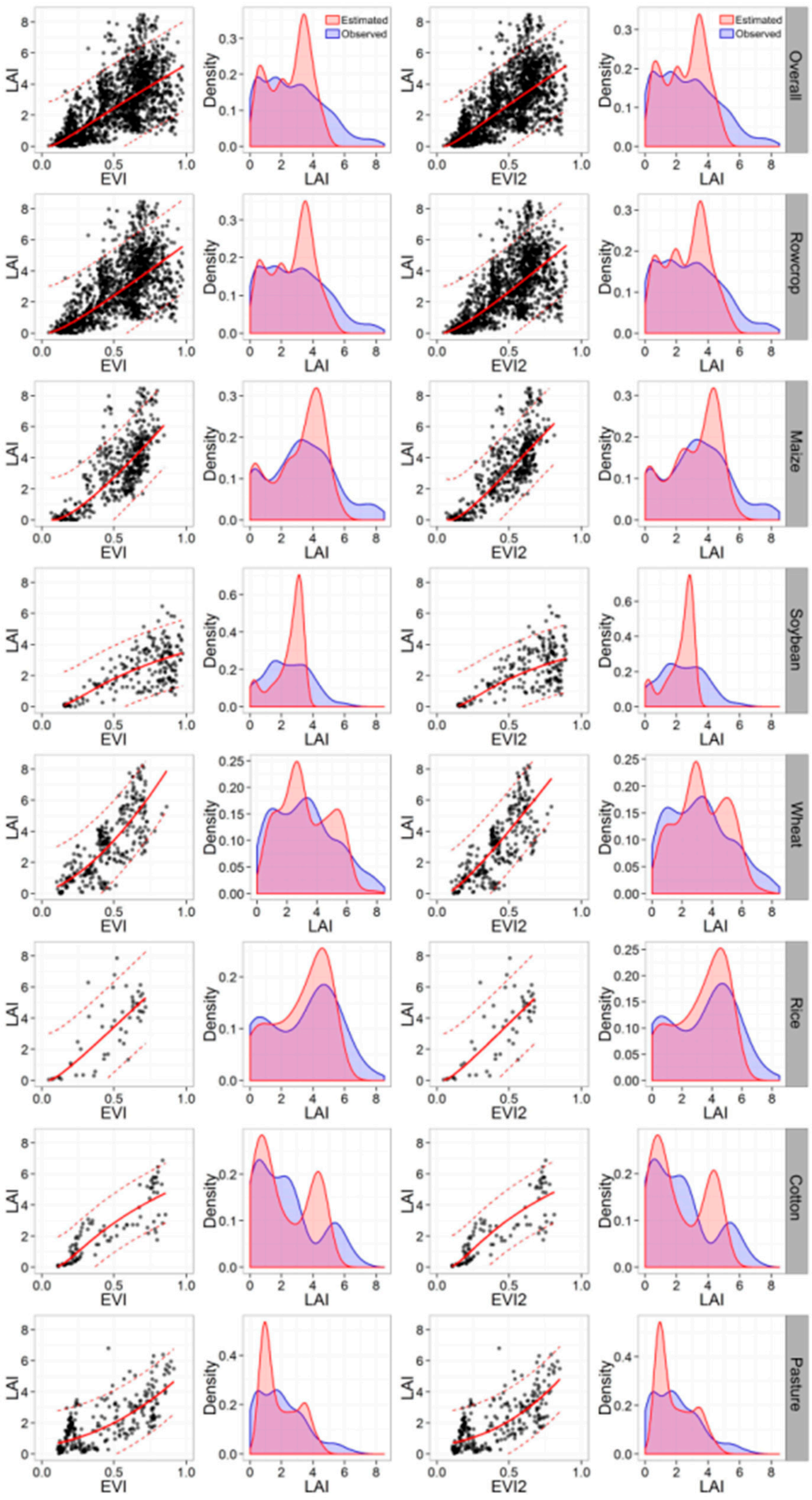

Figure S8. LAI-EVI and LAI-EVI2 relationships based on Theil-Sen regression and the density distributions of the measured and predicted LAI for the full-range dataset. The first and third columns show scatter plots between LAI and EVI/EVI2 as well as the relationship (solid red line) and prediction interval (dashed red line) based on Theil-Sen regression. The second and fourth columns show density distributions of the measured (blue) and predicted (red) LAI based on EVI and EVI2 respectively.

6. Analysis of the Effect of Sun-Sensor Geometry on LAI-VI Relationships

We assessed the effect of Sun-sensor geometry on the global LAI-VI relationships. Since all the Landsat TM and ETM+ data we used are nadir viewing images, the bidirectional reflectance is only affected by the Sun illumination geometry, which eliminates the hot-spot in BRDF. In Figure S9, we plotted the Sun illumination angles of all the Landsat data we used in this study in a polar coordinate. The sun illumination angles do not have a large variation, as the zenith angle is mostly within 25° to 60°, and the azimuth angle is between 50° to 150°. Figure S10 shows the residuals from the global LAI-EVI relationship of all samples plotted over zenith and azimuth angles. For each angle, the distribution of residuals is centered at zero and spread equally towards positive and negative space, which confirms a normal distribution assumption of the regression and indicates that there is no significant effect of the sun illumination angles on the residuals and the LAI-VI relationships.

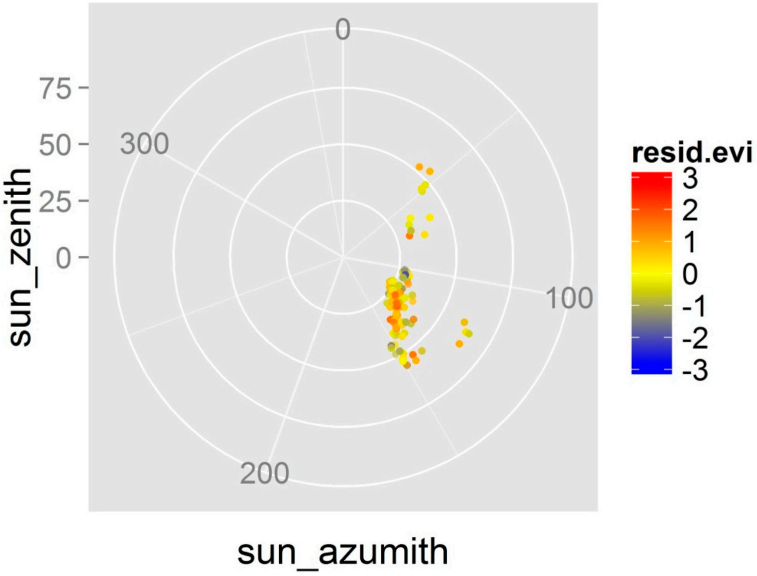

Figure S9. Distribution of the sun illumination angles of all the Landsat data used in this study in a polar coordinate.

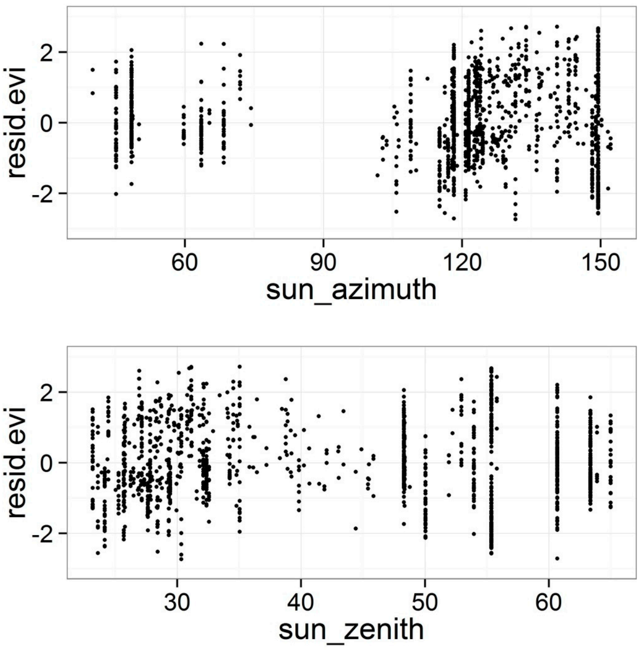

Figure S10. The residuals from global LAI-EVI relationship plotted over sun azimuth and zenith angles.

References

1. Gower, S.; Kirschbaum, A.A. *Big Foot Field Data for North American Sites, 1999–2003*; Oak Ridge National Laboratory Distributed Active Archive Center: Oak Ridge, TN, USA, 2088; doi:10.3334/ORNLDAAC/868. Available online: http://www.daac.ornl.gov (accessed on 10 October 2012).
2. Verma, S.B.; Dobermann, A.; Cassman, K.G.; Walters, D.T.; Knops, J.M.; Arkebauer, T.J.; Suyker, A.E.; et al. Annual carbon dioxide exchange in irrigated and rainfed maize-based agroecosystems. *Agric. For. Meteorol.* **2005**, *131*, 77–96, doi:10.1016/j.agrformet.2005.05.003.
3. Jones, J.W.; Hoogenboom, G.; Porter, C.H.; Boote, K.J.; Batchelor, W.D.; Hunt, L.A.; Wilkens, P.W.; Singh, U.; Gijsman, A.J.; Ritchie, J.T. The DSSAT cropping system model. *Eur. J. Agron.* **2003**, *18*, 235–265, doi:10.1016/S1161-0301(02)00107-7.
4. Mauney, J.R.; Lewin, K.F.; Hendrey, G.R.; Kimball, B.A. Growth and yield of cotton exposed to free-air CO_2 enrichment (FACE). *Crit. Rev. Plant Sci.* **1992**, *11*, 213–222, doi:10.1080/07352689209382341.
5. Mauney, J.R.; Kimball, B.A.; Pinter, P.J., Jr.; LaMorte, R.L.; Lewin, K.F.; Nagy, J.; Hendrey, G.R. Growth and yield of cotton in response to a free-air carbon dioxide enrichment (FACE) environment. *Agric. For. Meteorol.* **1994**, *70*, 49–67, doi:10.1016/0168-1923(94)90047-7.
6. Kim, S.H.; Hong, S.Y.; Sudduth, K.A.; Kim, Y.; Lee, K. Comparing LAI Estimates of Maize and Soybean from Vegetation Indices of Multi-resolution Satellite Images. *Korean J. Remote Sens.* **2012**, *28*, 597–609, doi:10.7780/kjrs.2012.28.6.1.
7. Anderson, M. *SMEX02 Regional Vegetation Sampling Data, Iowa*; Digital Media; National Snow and Ice Data Center: Boulder, CO, USA, 2003.
8. Bosch, D.D.; Marshall, L.; Rowland, D.; Jacobs, J. *SMEX03 Vegetation Data: Georgia*; Digital Media; National Snow and Ice Data Center: Boulder, CO, USA, 2004.
9. Jackson, T.J.; McKee, L. *SMEX03 Vegetation Data: Oklahoma*; Digital media; National Snow and Ice Data Center: Boulder, CO, USA, 2004.
10. Validation of Land European Remote Sensing Instruments (VALERI). Available online: http://w3.avignon.inra.fr/valeri/ (accessed on 10 October 2012).
11. Earth Observation Campaigns Data (Provided by European Space Agency). Available online: https://earth.esa.int/web/guest/campaigns (accessed on 2 September 2013).
12. Kutsch, W.L.; Aubinet, M.; Buchmann, N.; Smith, P.; Osborne, B.; Eugster, W.; Wattenbach, M.; Schrumpf, M.; Schulze, E.D.; Tomelleri, E.; et al. The net biome production of full crop rotations in Europe. *Agric. Ecosyst. Environ.* **2010**, *139*, 336–345, doi:10.1016/j.agee.2010.07.016.
13. AsiaFlux. Available online: http://www.asiaflux.net/index.html (accessed on 10 October 2012).
14. Yi, Y.; Yang, D.; Huang, J.; Chen, D. Evaluation of MODIS surface reflectance products for wheat leaf area index (LAI) retrieval. *ISPRS J. Photogramm. Remote Sens.* **2008**, *63*, 661–677, doi:10.1016/j.isprsjprs.2008.04.004.
15. Peischl, S.; Walker, J.P.; Rüdiger, C.; Ye, N.; Kerr, Y.H.; Kim, E.; Bandara, R.; Allahmoradi, M. The AACES field experiment: SMOS calibration and validation across the Murrumbidgee River catchment. *Hydrol. Earth Syst. Sci.* **2012**, *16*, 1697–1708, doi:10.5194/hess-16-1697-2012.
16. Panciera, R.; Walker, J.; Kalma, J.D.; Kim, E.J.; Hacker, J.M.; Merlin, O.; Berger, M.; Skou, N. The NAFE’05/CoSMOS data set: Toward SMOS soil moisture retrieval, downscaling, and assimilation. *IEEE Trans. Geosci. Remote Sens.* **2008**, *46*, 736–745, doi:10.1109/TGRS.2007.915403.
17. Merlin, O.; Walker, J.P.; Kalma, J.D.; Kim, E.J.; Hacker, J.; Panciera, R.; Young, R.; Summerell, G.; Hornbuckle, J.; Hafeez, M.; et al. The NAFE’06 data set: Towards soil moisture retrieval at intermediate resolution. *Adv. Water Resour.* **2008**, *31*, 1444–1455, doi:10.1016/j.advwatres.2008.01.018.
18. Panciera, R.; Walker, J.; Jackson, T.; Gray, D. A.; Tanase, M. A.; Ryu, D.; Monerris, A.; Yardley, H.; Rudiger, C.; Wu, N; et al. The Soil Moisture Active Passive Experiments (SMAPEx): Toward soil moisture retrieval from the SMAP mission. *IEEE Trans. Geosci. Remote Sens.* **2013**, *52*, 1–18, doi:10.1109/TGRS.2013.2241774.
19. Chen, J.M. Optically-based methods for measuring seasonal variation of leaf area index in boreal conifer stands. *Agric. For. Meteorol.* **1996**, *80*, 135–163, doi:10.1016/0168-1923(95)02291-0.
20. Cohen, W.B.; Maiersperger, T.K.; Gower, S.T.; Turner, D.P. An improved strategy for regression of biophysical variables and Landsat ETM+ data. *Remote Sens. Environ.* **2003**, *84*, 561–571.
21. Viña, A.; Gitelson, A.A.; Nguy-Robertson, A.L.; Peng, Y. Comparison of different vegetation indices for the remote assessment of green leaf area index of crops. *Remote Sens. Environ.* **2011**, *115*, 3468–3478, doi:10.1016/j.rse.2011.08.010.

22. Nguy-Robertson, A.; Gitelson, A.; Peng, Y.; Viña, A.; Arkebauer, T.; Rundquist, D. Green Leaf Area Index Estimation in Maize and Soybean: Combining Vegetation Indices to Achieve Maximal Sensitivity. *Agron. J.* **2012**, *104*, 1336–1347, doi:10.2134/agronj2012.0065.
23. Gitelson, A.A. Remote estimation of canopy chlorophyll content in crops. *Geophys. Res. Lett.* **2005**, *32*, L08403, doi:10.1029/2005GL022688.
24. Gitelson, A.A. Remote estimation of leaf area index and green leaf biomass in maize canopies. *Geophys. Res. Lett.* **2003**, *30*, 1248, doi:10.1029/2002GL016450.
25. Gitelson, A.A.; Gritz, Y.; Merzlyak, M.N. Relationships between leaf chlorophyll content and spectral reflectance and algorithms for non-destructive chlorophyll assessment in higher plant leaves. *J. Plant Physiol.* **2003**, *160*, 271–282, doi:10.1078/0176-1617-00887.

© 2016 by the authors; licensee MDPI, Basel, Switzerland. This article is an open access article distributed under the terms and conditions of the Creative Commons by Attribution (CC-BY) license (http://creativecommons.org/licenses/by/4.0/).
